# 1,4-Bis(Acylhydrazone)-Based Polycatenar Liquid Crystals: Self-Assembly, Molecular Switching, and Gelation Properties

**DOI:** 10.1021/acsomega.5c00974

**Published:** 2025-05-23

**Authors:** Wilson Aparecido de Oliveira, Mohamed Alaasar, Yu Cao, Eduard Westphal

**Affiliations:** † Departamento Acadêmico de Química e Biologia, Universidade Tecnológica Federal do Paraná, Curitiba 81280-340, Brazil; ‡ Department of Chemistry, 28117 Universidade Federal de Santa Catarina, Florianópolis 88040-900, Brazil; § Institute of Chemistry, 9176 Martin Luther University Halle-Wittenberg, 06120 Halle, Germany; ∥ Department of Chemistry, Faculty of Science, Cairo University, 12613 Giza, Egypt; ⊥ Shaanxi International Research Center for Soft Matter, State Key Laboratory for Mechanical Behavior of Materials, 12480 Xi’an Jiaotong University, Xi’an 710049, P. R. China

## Abstract

Photochromic materials allow their properties to be reversibly modulated by using light, offering broad applicability and significant scientific interest. In the case of liquid crystals (LCs), this opens up possibilities for controlling the self-organization, its properties, and even extinguishing mesomorphism. Among the diverse liquid crystalline functional groups, acylhydrazones remain the underexplored photochromic group despite their advantageous characteristics, such as ease of synthesis, versatility, reversible *E-Z* photoisomerization, and gelation. Building on efforts to demonstrate the versatility of this group, this study introduces, for the first time, a second acylhydrazone unit into linear polycatenar molecules, enabling a systematic investigation of the effects of structural variations, including the number of peripheral alkoxy chains, the orientation of the acylhydrazone group, and expansion of the rigid core. These modifications led to the synthesis of six new molecules, whose thermal, mesomorphic, switching, and gelation properties were systematically investigated. The results demonstrated that the introduction of a second acylhydrazone unit significantly enhanced these properties, with 5 molecules showing LC properties. Depending on the molecular design, mesomorphism was stabilized, and self-organization varied between hexagonal columnar (Col_h_) and bicontinuous cubic (Cub_bi_) phases. The orientation of the acylhydrazone units influenced mesomorphism, drastically altered the photoisomerization rate, and induced mild luminescence. These properties were correlated with intermolecular interactions and the way they promoted self-organization in the respective mesophases. Additionally, the two molecules formed stable, photoisomerizable gels, highlighting the versatility of acylhydrazone units in the development of multifunctional materials.

## Graphical abstract

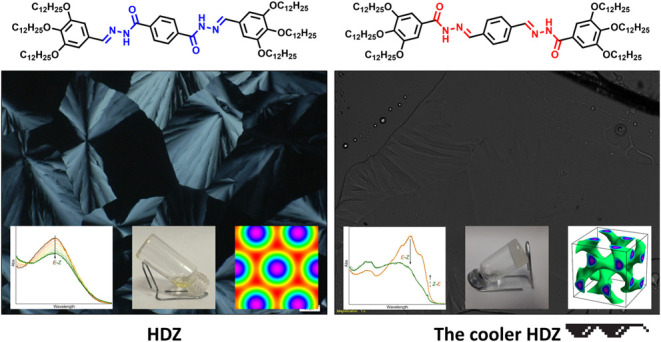

## 1 Introduction

The ability to control microscopic systems at the macroscopic level has long been a scientific aspiration. While significant advancements have been made with molecular switches and machines, there remains considerable potential for further improvement in these systems.^1–3^ A widely used approach for controlling material properties involves the use of light, achieved through photoresponsive materials.^4–6^ Molecular moieties commonly employed in photoresponsive materials include coumarins,^7–9^ porphyrins,^10^ and azobenzenes.^11–13^ Azobenzenes, in particular, are extensively used in the preparation of photoresponsive liquid crystals (LCs),^14–16^ enabling the control of mesomorphism disruption,^17^ phase changes, and even isothermal chirality switching.^18^ Another class known for its photoisomerization properties is acylhydrazones, which present good *E-Z* photoisomerization efficiency, stability, and reliability, and whose isomerization rate can be tailored—either accelerated or slowed—by modifying the functional groups attached to their aromatic rings.^19,20^ Additionally, this class is recognized for forming stable gels through van der Waals interactions, hydrogen bonds, π-π interactions, and responsiveness to physical and chemical stimuli.^21–24^

Although much less explored than azobenzenes, some examples of LC molecules containing acylhydrazones have already been reported in the literature. In 2011, Tschierske’s group identified acylhydrazones as promising candidates for room-temperature LCs exhibiting a Col_h_ mesophase.^25^ Guo et al. reported wide ranges of columnar phases in acylhydrazone-based gallic trimers and tetramers.^26^ Singh and collaborators have examined the effect of small structural changes on the mesomorphism of acylhydrazone derivatives, including the addition of a metal, the introduction of an amide group, and varying the number of substituents on one side of the molecule.^27–29^ With the same focus, our group also reported the viability of acylhydrazones in polycatenar LCs and how terminal chains in different positions interfere with photoisomerization.^30^ More recently, Mali et al. published the combination of acylhydrazones with cinnamate esters to obtain calamitic LCs with a terminal pyridine, investigating the effect of chain length on such systems and pyridine orientation.^31^ However, to date, the expansion of the mesogenic core through the incorporation of a second acylhydrazone unit and its impact on the combination of LC behavior, photoresponsiveness, and gelation properties have not been explored.

Therefore, to address this gap and enhance the understanding and versatility of acylhydrazones in LC research, we report herein the influence of integrating a second acylhydrazone unit into linear polycatenar molecules. We also explored how the orientation of the photochromic group influences thermal, photophysical, liquid crystalline, and gelation properties, as well as the impact of varying the number of terminal chains.

## 2 Results and Discussion

To carry out the proposed studies, six new polycatenar molecules were designed (Figure 1), each containing two or three long alkoxy chains on each side along with two acylhydrazone units positioned in a 1,4-relationship. The targeted structures also allowed for the inversion of the orientation of the photochromic units, as well as the expansion of the rigid core, enabling a systematic investigation.

**Figure 1:**
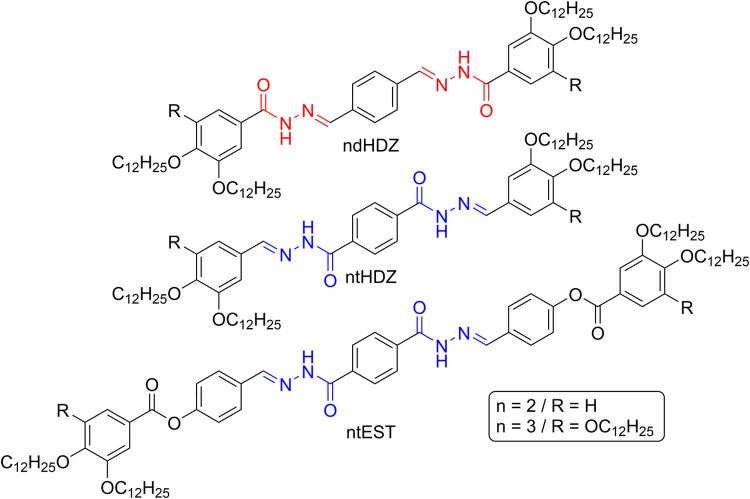
General chemical structures of the newly synthesized acylhydrazones under discussion (*n* refers to the number of alkoxy chains on each side of the molecule).

All nomenclature used are standardized with the formula **nxY** where “**n**” stands for the number of long aliphatic chains; “**x**” indicates the orientation of the acylhydrazone isomer, with “**d**” referring to derivates of terephthalaldehyde and “**t**” to those derived from terephthalhydrazyde; “**Y**” distinguish if the chains are directly connected to the acylhydrazone (**HDZ**) or connected via a phenylester spacer (**EST**), resulting in the expanded molecule.

### 2.1 Synthesis

In general, all of the molecules were synthesized according to Scheme 1, with a good yield and high purity, confirmed by ^1^H Nuclear Magnetic Resonance (NMR) and Elemental Analysis. ^13^C NMR analysis could not be performed for all samples due to their low solubility in the available solvents and conditions compatible with NMR. In order to improve solubility and also break the molecular aggregation, which strongly broadened the NMR peaks and hindered interpretation, DMSO-*d*_6_ was used in some samples. Detailed synthetic routes, experimental procedures, and analytical data can be found in Supporting Information. It is important to highlight that molecules containing only 1 chain on each side (**1dHDZ** and **1tHDZ**) were also synthesized. However, their extreme insolubility and very high melting temperatures prevented reliable characterizations, and consequently, they were not investigated further.

**Scheme 1:**
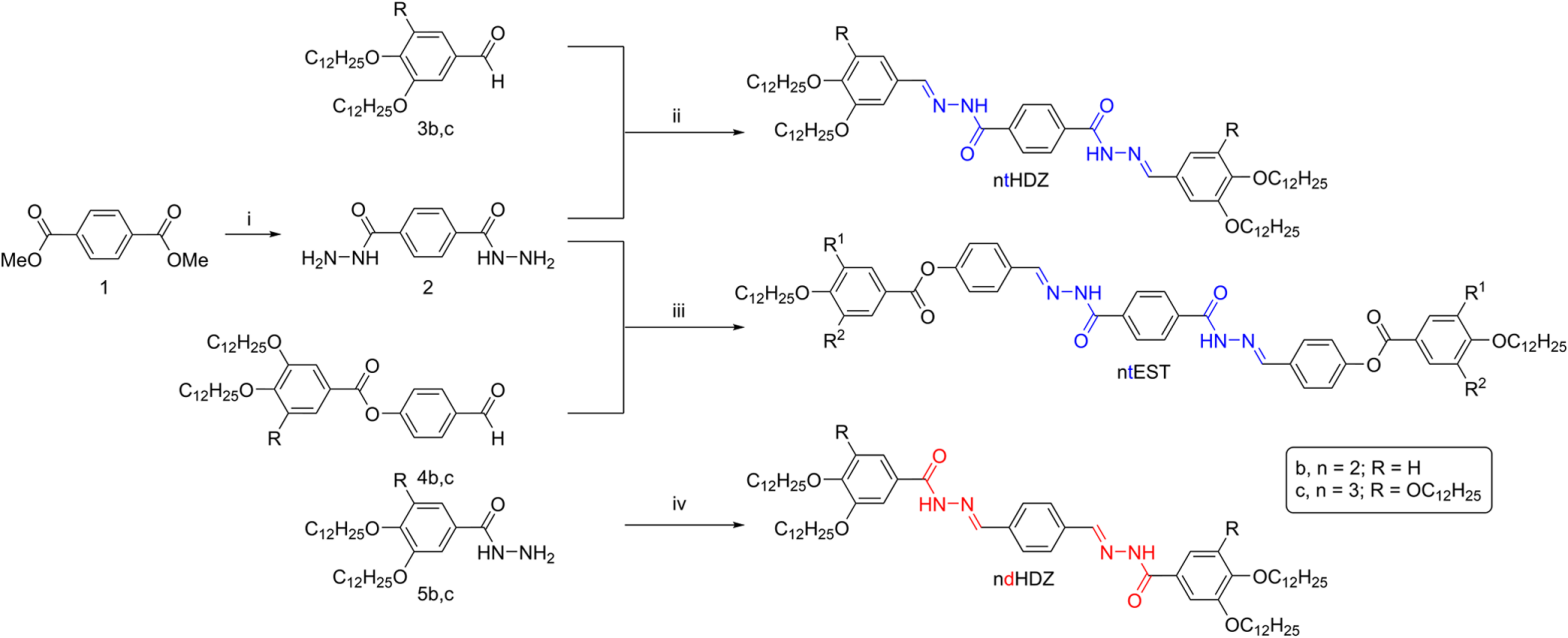
Synthetic Route for Intermediaries and Target Compounds: (i) NH_2_NH_2_·H_2_O and Toluene; (ii) Ethanol and Catalytic CH_3_COOH or CF_3_COOH; (iii) 1,2-dichlorothane and Catalytic CH_3_COOH or CF_3_COOH; (iv) Terephthalaldehyde and Catalytic CH_3_COOH

### 2.2 Characterization

All planned molecules had their thermal stability and phase transitions investigated by Polarized Optical Microscopy (POM), Differential Scanning Calorimetry (DSC), and Thermogravimetric Analysis (TGA). Those that showed liquid crystalline behavior had the mesophase further characterized via X-ray diffraction (XRD), using both wide-angle and small-angle X-ray scattering (WAXS and SAXS, the latter with synchrotron radiation). The results are summarized in Tables 1 and 2.

**Table 1:** Transition Temperatures, Associated Enthalpy Values, and Decomposition Temperatures for the Synthesized Compounds^a^

Molecule	***T*/°C** [Δ*H*/kJ mol^–1^]^b^	*T*_dec_ /°C^c^
	Heating	
**2dHDZ**	Cr **213** [74.2] (Col_h_ **207** [2.5]) Iso	**283**
**3dHDZ**	Cr **143** [5.8] Cub_bi_ **173** [0.4] Iso	**286**
**2tHDZ**	Cr **216** [99.7] Col_h_ **224** [2.4] Iso	**306**
**3tHDZ**	Cr **157** [10.6] Col_h_ **186** [3.2] Iso	**311**
**2tEST**	Cr **147** [21.7] Cr′ **278** [40.7] Iso	**274**
**3tEST**	Cr **181** [74.5] Col_h_ **212** [2.8] Iso	**276**

a Abbreviations: Cr = crystalline state, Iso = isotropic liquid, Col_h_ = hexagonal columnar mesophase, Cub_bi_ = Cubic bicontinuous mesophase, () = monotropic phase.

b Determined by DSC (peak temperatures) during the second heating cycle using a rate of 10 °C min^–1^.

c Determined by TGA measurements under nitrogen atmosphere with a heating rate of 10 °C min^–1^. The values refer to the temperature at which 1% of mass was lost by the material. A complete table with cooling can be found in the SI. A complete table, including cooling, can be found in the SI (Table S1).

**Table 2:** WAXS and SAXS Data and Lattice Parameters for the Liquid Crystalline Target Compounds^a^

compound	mesophase (*T*/°C) lattice parameters	Miller indices (*hkl*)	*d*_obs_/nm	*d*_cal_/nm
**2dHDZ** (WAXS)	Col_h_ (205) *a* = 3.55 nm	10	3.06	3.06
		11	1.76	1.77
		20	1.56	1.53
		*diff*	0.47	
**3dHDZ** (WAXS)	Cub_bi_ (155) (Ia3d) *a* = 7.85 nm	211	3.19	3.20
		220	2.77	2.77
		321	2.08	2.10
		400	1.98	1.96
		332	1.67	1.67
		422	1.63	1.60
		440	1.37	1.39
		*diff*	0.46	
**2tHDZ** (WAXS)	Col_h_ (195) *a* = 3.65 nm	10	3.17	3.18
		21	1.22	1.20
		31	0.86	0.88
		41	0.70	0.69
		*diff*	0.46	
**3tHDZ** (SAXS)	Col_h_ (175) *a* = 3.57 nm	10	3.089	3.088
		11	1.782	1.783
		20	1.544	1.544
**3tEST** (SAXS)	Col_h_ (190) *a* = 4.76 nm	10	4.123	4.122
		11	2.380	2.380
		20	2.061	2.061
		21	1.558	1.558

a Abbreviations: *a* = lattice parameter.

The thermal stability of the products (*T*_dec_) was determined by TGA measurements under a nitrogen atmosphere, being considered the temperature at which 1% of mass was lost by the material. All the compounds investigated exhibited decomposition at temperatures higher than 270 °C, indicating good thermal stability. Among the different types of molecules slight dependence of the structure on thermal stability was observed, where compounds with four alkoxy chains exhibited slightly lower stability. Furthermore, while the **ntHDZ**-type has the highest decomposition temperature, their ester analogous **ntEST** had the lowest temperature, demonstrating a loss in stability with the ester groups.

As summarized in Table 1, **2dHDZ** exhibits a monotropic LC phase as indicated by the single high-enthalpy transition on heating (74 kJ mol^–1^), while two transitions are observed on cooling (see Figure S2a in the SI for the DSC traces), accompanied by the appearance of a discotic fan-texture with homeotropic aligned regions (Figure 2a). These textural observations are characteristic for the hexagonal columnar mesophase (Col_h_), and therefore, it was assigned as the Col_h_ phase, which was further confirmed by XRD investigations (Section 2.3). Increasing the number of terminal chains from 2 to 3 (on each end) lowered all transition temperatures, resulting in an enantiotropic LC phase instead of the monotropic one observed for **2dHDZ**. Under POM, the mesophase of **3dHDZ** was initially challenging to observe as no distinct texture was visible. Only faint grooves, likely representing domains, could be discerned, particularly when the intensity of the incident light was increased and/or the polarizers were slightly uncrossed (Figure 2b). This phase is characterized also by its high viscosity, and a reversible phase transition was recorded on DSC heating and cooling scans (see Figure S2b). These observations suggest the assignment of this mesophase as a cubic phase, as confirmed also by XRD investigations (Section 2.3). Although cubic mesophases are not among the most commonly observed in thermotropic liquid crystals and have not yet been reported for acylhydrazone derivatives, several studies have documented their occurrence in liquid crystals, and their occurrence has been documented in both lyotropic^32^ and thermotropic systems, encompassing a wide range of structures such as ionic compounds,^33,34^ dendrimers,^35^ giant molecules,^36^ and polymers.^37^ Moreover, different types of cubic organizations have already been demonstrated, including micellar,^35^ bicontinuous cubic,^38,39^ and triple network structures,^40,41^ among others.^42^ In the case of polycatenar LCs, this also holds true for symmetric and nonsymmetric terminal chains, often resulting in wide temperature ranges.^43–48^ Due to their isotropic molecular organization, mesophases of this type typically lack birefringence, making them difficult to observe using POM (Figure S3).

**Figure 2:**
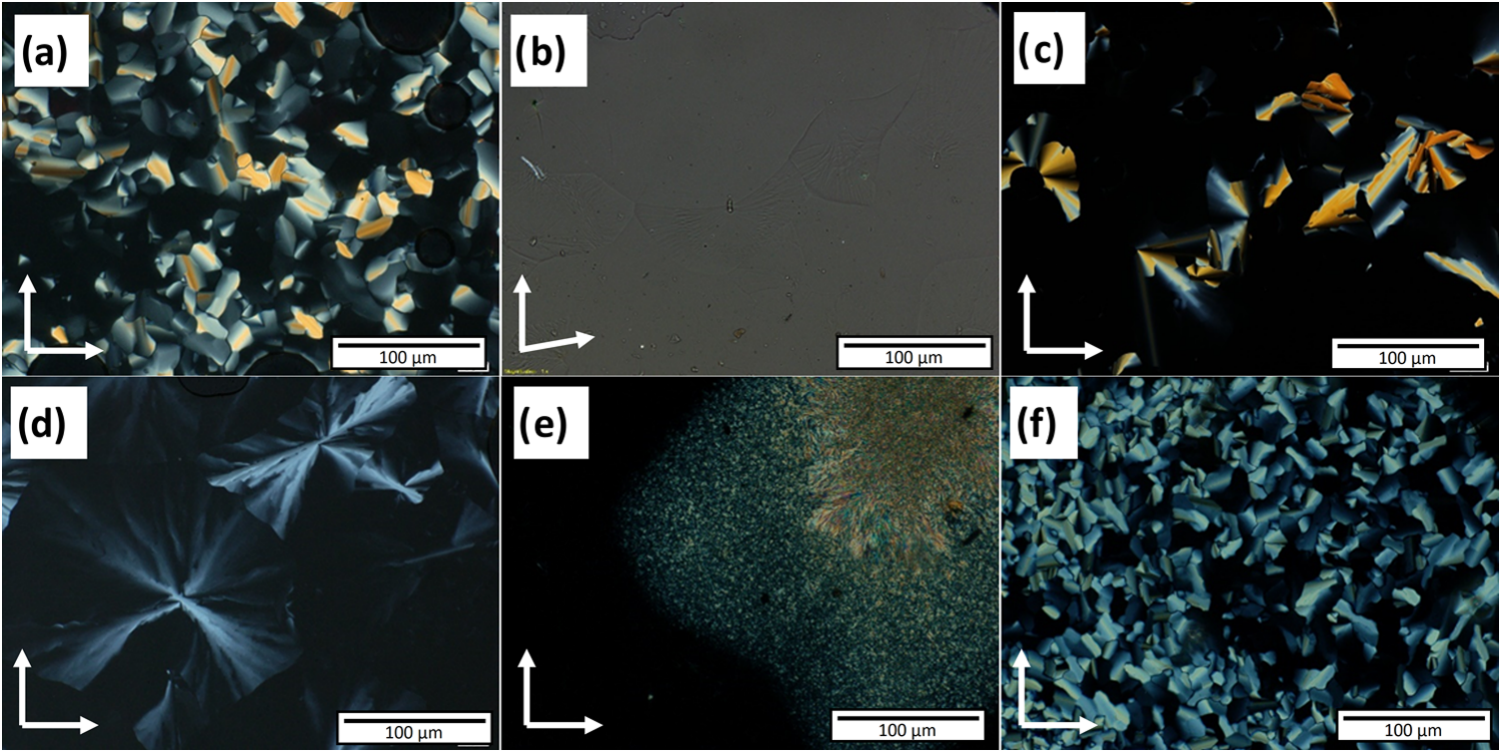
Textures observed by POM for the synthesized compounds, on cooling from isotropic liquid, crossed polarizers in all images except (b) (direction of polarizers is indicated by white arrows): (a) fan-texture of **2dHDZ** at 205 °C; (b) grooves observed for **3dHDZ** at 170 °C (10° uncrossed polarizers); (c) fan-texture of **2tHDZ** at 213 °C; (d) fan-texture observed for the molecule **3tHDZ** at 180 °C; (e) crystallization of **2tEST** at 230 °C; (f) fan-texture of **3tEST** at 200 °C.

The inversion in the direction of the acylhydrazone units, from **ndHDZ** to **ntHDZ**, despite maintaining the shape of the molecule, promoted small changes in the thermal properties of the compounds. For **2tHDZ**, the Col_h_ mesomorphism is stabilized (Figure 2c), becoming enantiotropic (Figure 2c in the SI), with a slightly higher mesomorphism range on cooling. Similar to the case for the **ndHDZ**-type, the increase in the number of chains (**3tHDZ**) lowered the transition temperatures and stabilized the mesomorphism (Figure 2d in SI), in this case favoring a Col_h_ self-organization, as evidenced by the distorted fan-texture (Figure 2d).

Analyzing both types shows that the acylhydrazone direction impacts intermolecular interactions so that *t*-type compounds exhibit stronger interaction, evidenced by slightly higher transition temperatures and lower solubility (Table 1). On the other hand, increasing the number of terminal chains led to a significant decrease in the melting temperature and its associated enthalpy (Table 1) (≈70 kJ mol^–1^). The significant reduction in Δ*H* values, particularly during fusion, may suggest that the additional chain reduces the crystallinity of the solid, requiring less energy to transition to the liquid crystalline phase. Furthermore, the additional chains considerably increase the system’s entropy, which consequently leads to a decrease in transition temperatures.^49^ On the other hand, the increase in the number of chains also led to a broadening of the terminal regions of the molecules without modifying the central aromatic core, which can result in certain changes in the molecular packing of polycatenar molecules^50^ and, in the present case, stabilized the mesomorphism. For the **ndHDZ** series, the greater difference between the volumes of the terminal parts relative to the central molecular part induced a change in the mesomorphism from Col_h_ to Cub_bi_. Meanwhile, for the **ndHDZ** series, it resulted in only an increase in the mesophase range. However, when the molecular anisometry increased, as will be discussed below for the **ntEST** series, Col_h_ mesomorphism was formed and stabilized.

Although an increase in molecular anisometry generally favors mesomorphism, this was not observed for **2tEST**, where the expansion of the rigid core completely suppressed mesophase formation. DSC scans of **2tEST** (Figure S2e in SI) show two phase transitions during heating and cooling; however, these correspond only to Cr–Cr′ transitions, with the product melting directly into the isotropic liquid state at around 278 °C. Additionally, POM observations revealed no material fluidity between the phase transitions and no characteristic LC textures during either heating or cooling. Instead, only direct crystallization from the liquid state was observed during cooling (Figures 2e and S2e). The larger size of the rigid core combined with intermolecular hydrogen bonding and the presence of two additional phenylester groups enhanced intermolecular interactions, significantly lowering the free energy of the solid state. As a result, the Cr-LC transition temperature is pushed above the Cr-Iso transition temperature, leading to the direct melting into the isotropic liquid.

However, for **3tEST**, the expansion of the rigid core led to an increase in transition temperatures compared to **3tHDZ**, while the higher number of alkyl chains reduced the temperatures relative to **2tEST** due to entropic effects of the addition chains and contributed to stabilizing the mesomorphic behavior. Upon analyzing **3tEST** under POM, a fan-texture was observed (Figure 2f), suggesting a columnar LC phase. As indicated in Table 1, compound **3tEST** also exhibits large enthalpies of phase transition and significant thermal hysteresis between the melting and crystallization temperatures (Figure S2f in the SI). These data indicate a favoring of attractive forces at the center of the molecule over the steric effect at the ends, which supports the increased transition temperatures of **3tEST** compared to those of **3tHDZ**. On the other hand, the steric effect of the greater number of terminal chains counterbalances the significant increase in intermolecular forces observed for **2tEST**.

### 2.3 XRD Investigations

To gain more information about the molecular organization in the mesophases and to confirm the thermal and mesomorphic behavior described by POM and DSC, measurements of WAXS and SAXS (with synchrotron radiation) were performed for all LC materials, and the data are summarized in Table 2.

Upon analyzing the WAXS data of **2dHDZ** (Figure S5), three distinct peaks in the low angle regime were observed, which can be indexed as *d*_10_ (3.06 nm), *d*_11_ (1.76 nm), and *d*_20_ (1.56 nm), confirming the two-dimensional hexagonal lattice of a Col_h_ phase. In the wide-angle regions, a very broad peak around 0.47 nm is related to the average lateral liquid-like distance between the disordered aliphatic chains and aromatic cores, which, together with the absence of sharp peaks in this region, suggests the fluidity of the material.^51,52^ From the average values of the diffraction peaks, the lattice parameter (*a*), which is equivalent to the diameter of a disk, was determined to be 3.55 nm, significantly smaller than the molecular length (*L*) of the most extended molecular conformation calculated by using ChemBio3D Ultra software (5.23 nm).

All other compounds that had a hexagonal columnar mesophase speculated by POM also had the Col_h_ phase confirmed by XRD analysis due to appropriate indexing of the observed peaks, as described in Table 2 and shown in Figures S4–S9. The lattice parameter for each compound was also calculated, and from the presented data for **2dHDZ** (*a* = 3.55 nm) and **2tHDZ** (*a* = 3.65 nm), it is noted that the inversion of the functional group does not significantly affect the size of the disk. This is somewhat expected, as the change in the direction of the acylhydrazones does not significantly affect the molecular size. However, it is interesting to note that the increase in the number of peripheral chains also does not have a great influence on the size of the disk, as can be seen by comparing **2tHDZ** (*a* = 3.65 nm) and **3tHDZ** (*a* = 3.57 nm). An increase in the disk diameter is observed only when the molecule is effectively elongated through the incorporation of ester groups, as demonstrated for **3tEST** (*a* = 4.76 nm). This is consistent and expected given that the mesogenic core of **3tEST** (*L* = 6.45 nm) is longer when compared to **3tHDZ** (*L* = 5.23 nm), and the difference in *a* values (1.19 nm) matches the difference in *L* values (1.22 nm). However, it is interesting to note that when calculating the ratio between *a*/*L*, which indicates how much smaller the disk size is compared to the molecular length, a value of 0.68 is obtained for **3tHDZ** and 0.74 for **3tEST.** The lower value found for **3tHDZ** may be due to a combination of factors, such as the interdigitation of the aliphatic chains, the fact that due to the high temperature the chains will not be in their most extended form,^53^ or the molecules may be tilted within each column, creating an angle between the column axis and the vector normal to the disk.^54^

To compare the mesophases before and after molecular elongation, electron density (ED) maps for compounds **3tHDZ** and **3tEST** were reconstructed using SAXS data (Figure 3a,b). In both cases, a hexagonal pattern is clearly observed. For the **3tEST** product (Figure 3b), a greater separation between the regions of high electron density (larger red region) is observed, which may indicate a lower interdigitation of the chains and, therefore, separation of the disks caused by the greater number of peripheral chains of the disk. This is in accordance with the lower *a*/*L* ratio calculated for **3tHDZ**, as described before.

**Figure 3:**
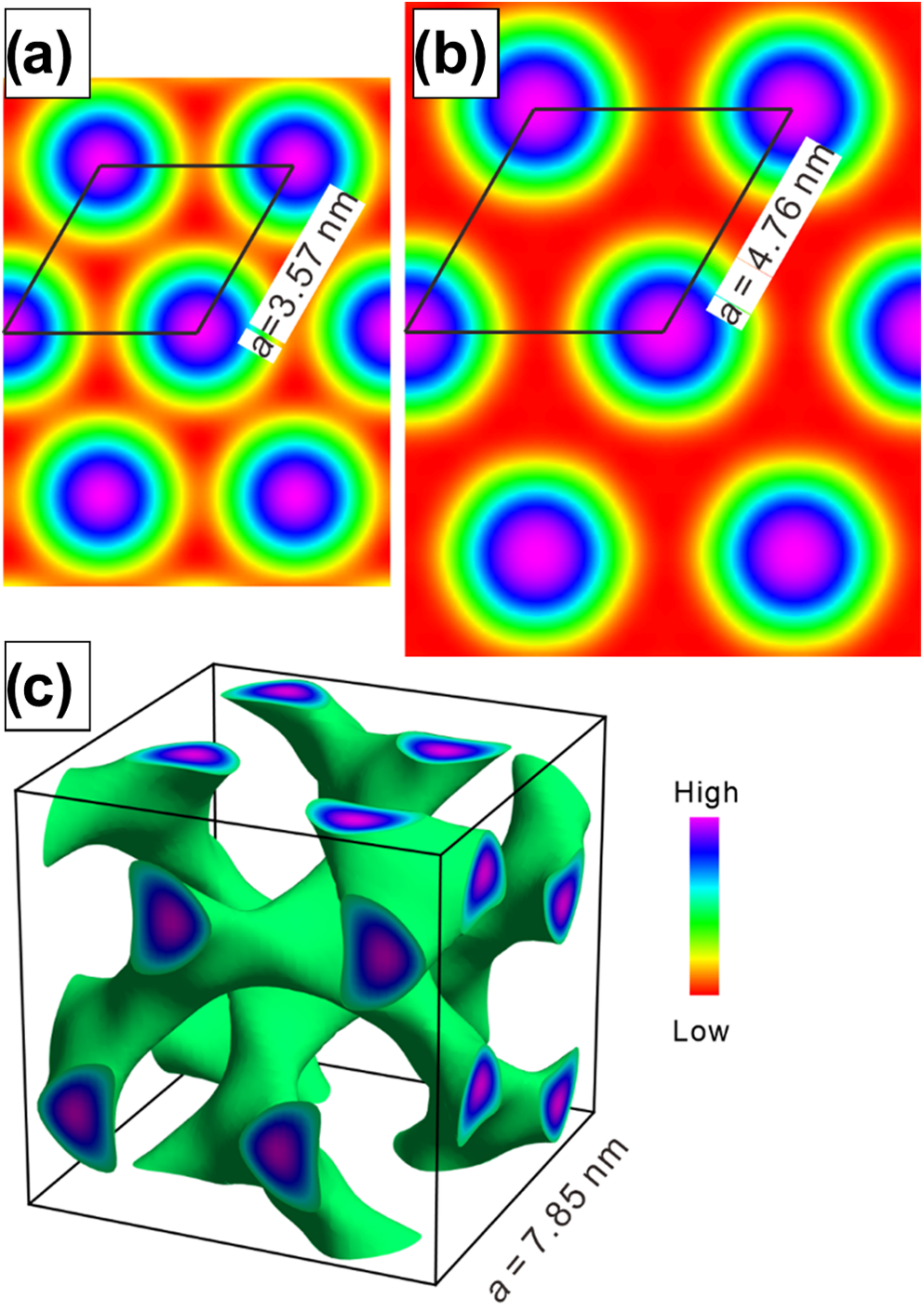
Reconstructed electron density map of the Col_h_ mesophase of compound **3tHDZ** (a) and **3tEST** (b) and the Cub_bi_ phase of **3dHDZ** (c). The lattice parameters are presented in each representation, and the figures were placed on a relative scale for a better comparison. Blue corresponds to high electron density, and red is the lowest.

For the **3dHDZ** molecule, which exhibits an isotropic texture by POM, WAXS data was collected (Table 2 and Figure S7), allowing the mesophase to be indexed as Bicontinuous Cubic (Cub_bi_) with *Ia*3̅*d* symmetry,^55,56^ which agrees with the observations under POM. Among the expected Bragg peaks, the following were observed in the diffractogram: *d*_211_ (strongest peak, √6), *d*_220_ (intense, √8), *d*_321_ (weak, √14), *d*_400_ (weak, √16), *d*_332_ (weak, √22), *d*_422_ (medium, √24), and *d*_440_ (weak, √32). Based on these observed diffraction peaks, the cell parameter *a* for this mesophase was calculated using the graph of 1/*d_hkl_* versus √(*h* ^2^ + *k* ^2^ + *l* ^2^) (Figure S8). The molecular organization of the cubic mesophase was also evaluated through the reconstruction of the electron density map, as demonstrated in Figure 3c. Aromatic cores are perpendicular to the double gyroid network and progress along it, as indicated by the high electron density region in the cubic lattice. The value found for *a* was 7.85 nm, which suggests the internetwork distance is 3.40 nm. The similar value compared to the lattice parameter of the 2D hexagonal phase suggests a similar degree of separation between the aromatic cores induced by the alkyl chains, which may result from the chains not adopting a fully extended conformation, back-folding, or interdigitation, or even from a combination of these effects.

### 2.4 Comparison with Related Molecules

Polycatenar compounds typically require at least four aromatic rings to stabilize mesomorphism.^50^ Interestingly, the acylhydrazones reported here possess only three rings, yet they already exhibit a diverse range of liquid crystalline behaviors. To provide a broader understanding of this phenomenon, we have also compared them with structurally similar molecules previously reported in the literature (Figure 4).^28,30,57^ Structurally similar molecules containing ester groups, which are incapable of forming intermolecular hydrogen bonds, do not exhibit polycatenar liquid crystalline behavior in their respective systems (**11** and **12**).^57^

**Figure 4:**
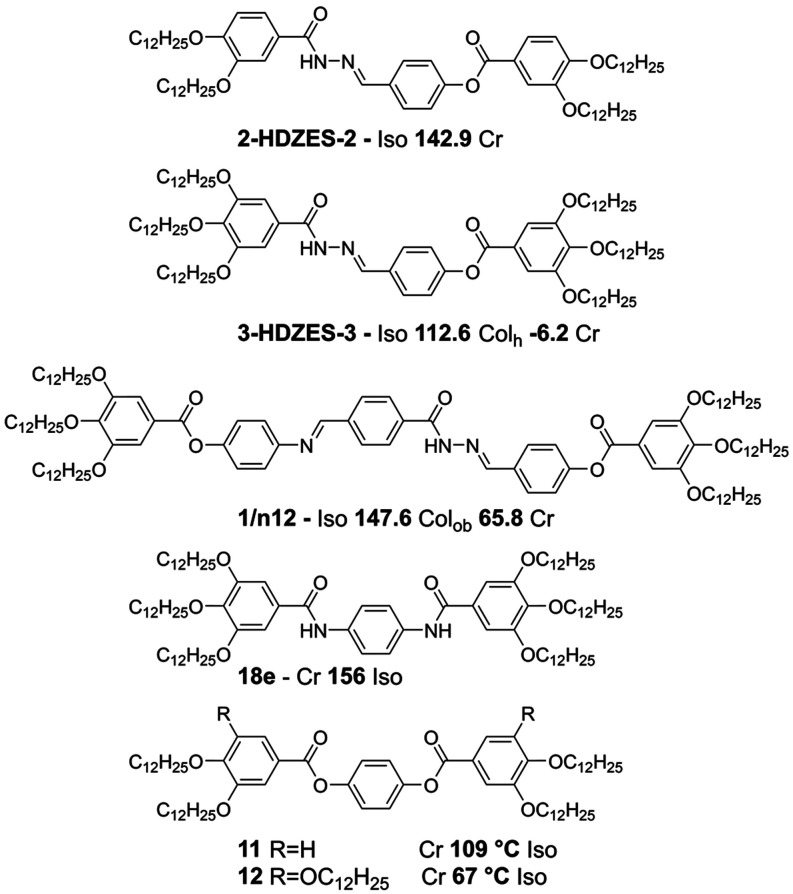
Molecular structures and phase transitions of related molecules **2-HDZES-2**, **3-HDZES-3**,^30^ **1/n12**,^28^ **18e**, **11**, **12**,^57^ found in the literature.

However, when one of the ester groups is replaced by an acylhydrazone unit, mesomorphism is stabilized—but only when six lateral chains are present (**3-HDZES-3**).^30^ Given that acylhydrazone units can form intermolecular hydrogen bonds,^58^ the observed mesomorphism can likely be attributed to the presence of these interactions. In contrast, compound **2-HDZES-2** exhibited a significantly higher melting temperature and the absence of mesophase formation despite the presence of hydrogen-bonding interactions.

The incorporation of a second acylhydrazone unit in place of the ester group further enhanced hydrogen bonding and strengthened intermolecular forces, leading to a significant increase in the transition temperatures. However, this modification also contributed to the stabilization of mesomorphism in both tetracatenar (**2tHDZ** and **2dHDZ**) and hexacatenar (**3tHDZ** and **3dHDZ**) molecules. Notably, this structural change also altered the molecular organization, shifting from a Col_h_ arrangement in **3-HDZES-3** to a Cub_bi_ phase in **3dHDZ**.

However, hydrogen bonding alone cannot fully explain this behavior, as compound **18e**,^57^ which contains two amide groups, did not exhibit any mesomorphism. Similarly, **2tEST**, despite having two acylhydrazone units, melted directly into the isotropic liquid, even though it is capable of forming hydrogen bonds and had a greater number of rings in the mesogenic core. Once again, in the last case, it is likely that the strong intermolecular interactions led to excessively high transition temperatures, ultimately favoring direct melting into the isotropic phase rather than stabilizing a mesophase.

Comparing the symmetric **3tEST** with the nonsymmetric **1/n12** molecule, where the difference lies in the exchange of an acylhydrazone for an imine group, it is noted that the **1/n12** exhibits a Col_ob_ mesophase, while the **3tEST** molecule presented a Col_h_ mesophase. Additionally, the incorporation of a second acylhydrazone significantly raised the clearing temperature and reduced the mesophase stability.

### 2.5 Photophysical Behavior

To evaluate the potential applicability of the synthesized molecules as photoresponsive materials, photoisomerization experiments were conducted using a UV–vis spectrometer and a fluorimeter (Figure 5). Acylhydrazones are known for their reversible photoisomerization process, with the *E* isomer generally exhibiting greater thermodynamic stability, except in cases where steric effects or intramolecular hydrogen bonding favor the *Z* isomer.^20^

**Figure 5:**
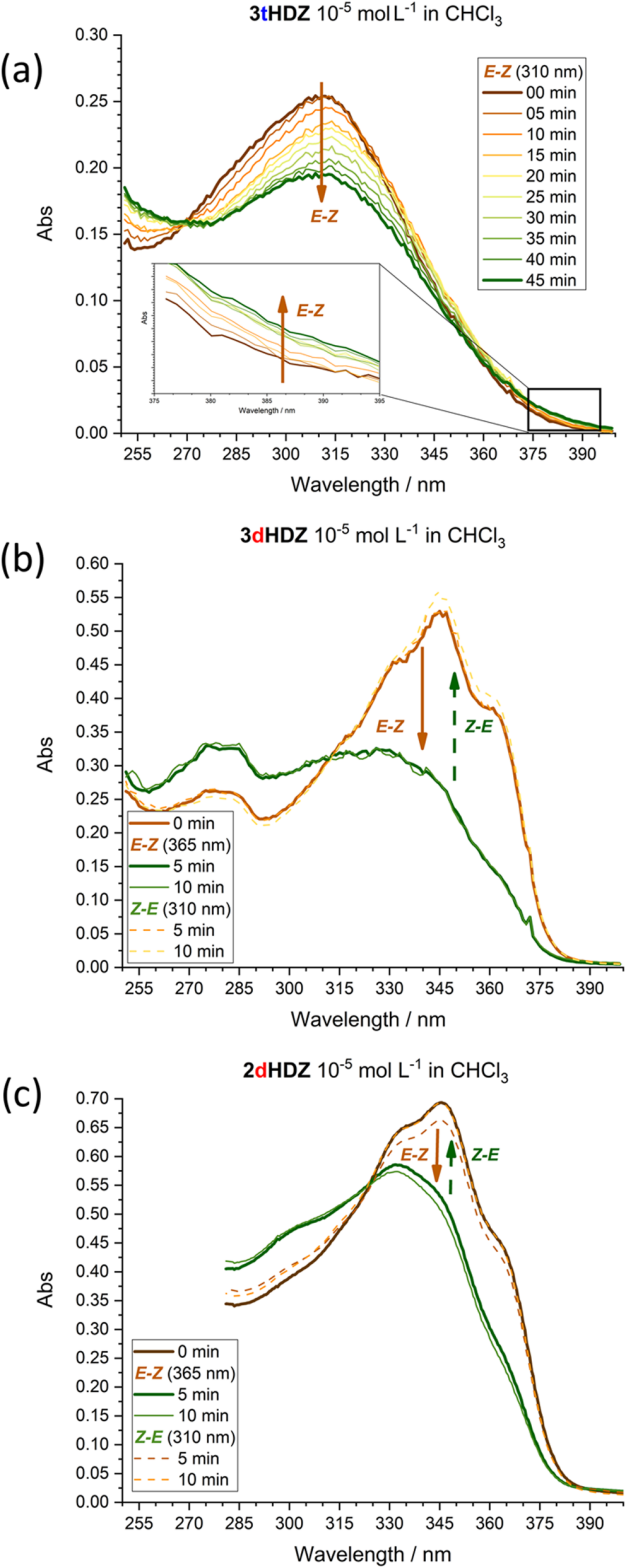
(a) Photoisomerization process for compound **3tHDZ** using a 310 nm lamp; Direct (*E-Z*) and reverse (*Z-E*) photoisomerization process for compounds **3dHDZ** (b) and **2dHDZ** (c) using lamps of different wavelengths. The solid brown arrow indicates the *E-Z* isomerization, while the dashed green arrow indicates the reverse process (*Z-E*).

In general, the *E* isomer exhibits a broad absorption band in the 300–330 nm region, corresponding to an allowed π-π* transition, which can be shifted beyond 360 nm depending on the substituents.^20,59^ The *Z* isomer, on the other hand, exhibits a low-intensity forbidden n-π* transition, so the photoisomerization can be monitored by the decrease in the intense *E* band and a slight increase in a band corresponding to the *Z* isomer.^19,20,59–61^ In some cases, this isomerization has also been observed by NMR and single-crystal analysis.^20,59^ For our compounds, a predominant absorption at 310 nm was observed for the **ntHDZ**-type, with a shift to 345 nm upon acylhydrazone inversion (**ndHDZ**-type) (Figures 5, S12 and Table S2).

When compound **3tHDZ** was exposed to 310 nm UV light (Figure 5a), a gradual decrease of the 310 nm band, related to the π-π* transition of the *E* isomer, was observed. Simultaneously, a low-intensity shoulder around 385 nm and a band near 250 nm, corresponding to the n-π* and π-π* transitions of the *Z* isomer, respectively, showed a slight increase in intensity. These changes indicate slow photoisomerization.

The spectrum obtained for **3dHDZ** (Figure 5b) exhibited distinct behavior, with the initial absorption maximum shifted to the 345 nm region. After 5 min of exposure to light with a wavelength of 365 nm, the material reached the photostationary state, evidenced by the decrease of the band at 345 nm and the increase of the bands at 280 and 310 nm. Additionally, no further changes were observed in the spectra even with continued exposure (up to 10 min). When the solution was exposed to light at 310 nm, the reverse photoisomerization process occurred, with the bands at 280 and 310 nm decreasing while the band at 345 nm increased. No significant changes were observed in the spectrum after continuing the exposure for up to 10 min. This indicates the reversibility of the photoisomerization, which occurred much faster than that of its isomer **3tHDZ**, only by inversion of the acylhydrazone groups.

The absorption spectrum of **2dHDZ** (Figure 5c) has an initial absorption maximum at 345 nm (π-π*). After 5 min of exposure to 365 nm light, a slight decrease in the band at 345 nm and an increase in the band at 300 nm (n-π*) were already noticeable, indicating photoisomerization. The process was repeated until a total exposure time of 10 min. The reverse photoisomerization process could also be observed using a 310 nm lamp. In this case, there was a decrease in the band at 300 nm and an increase in the band at 345 nm. The **2tHDZ**, on the other hand, did not undergo the photoisomerization process but exhibited luminescence in chloroform (Figure S10). Since the excited-state decay processes are competitive, this indicates that the orientation of the acylhydrazone group strongly influences the photoisomerization rate and the preferred relaxation from the excited-state mechanism.

For no material was back thermal relaxation (*Z* to *E*) at ambient temperature observed, given that for most acylhydrazones, this process is quite slow and may take days or even weeks to occur.^20,59^

The **2tEST** also showed indications of luminescence in ethanol solution (Figure S11), which decreases in intensity when using water as cosolvent and increases when using DCM as cosolvent. Meanwhile, the **3tEST** exhibited slow and nonphotoreversible photoisomerization (Figure S12).

We also conducted photoisomerization tests on the **2dHDZ** mesophase (Figure S13). This molecule was chosen due to its fast isomerization and birefringent mesophase. With the sample at 203 °C, after exposure to 365 nm light for 10 min, we observed the appearance of an isotropic region, while the region not exposed to light remained unchanged. However, the sample did not return to its previous state, either by exposure to 310 nm light or by maintaining heating for some minutes. The material exhibited mesomorphism only upon subsequent cooling, occurring at a temperature lower than that expected for the compound. Also, during the isomerization at high temperature, the solid turned yellowish. This behavior could also be attributed to partial photodecomposition of the material as we are favoring an excited state at such a very high temperature.

### 2.6 Gel Formation

During the purification stage of **3tEST**, upon heating a solution of this molecule in toluene and allowing it to cool to room temperature, a gel was formed (Figure S14). Acylhydrazones and liquid crystalline acylhydrazones are known for their ability to form molecular gels.^28,62–64^ Given this remarkable behavior, a controlled study was conducted to assess the formation of stable gels at room temperature with the other final compounds (Table S3). The tests were carried out in solvents such as heptane, toluene, chloroform, cyclohexane, and decane.

Besides **3tEST**, compound **3dHDZ** was able to form an unstable gel at high concentrations using cyclohexane as the solvent and under refrigeration; however, when the material was heated to room temperature, the gel fell apart. On the other hand, when the **2dHDZ** solution was prepared in toluene, it formed an opaque white gel (Figure 6a).

**Figure 6:**
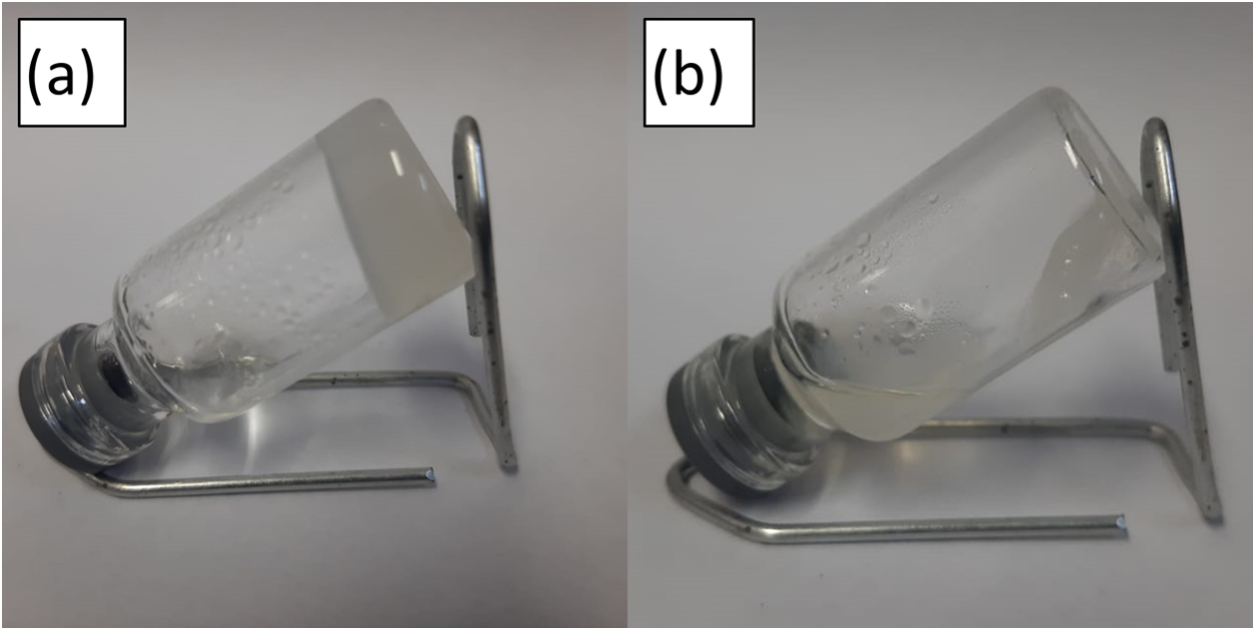
Photoisomerization tests in the gel for the **2dHDZ** molecule in toluene at 4 mg·mL^–1^: (a) gel before exposure to 310 nm radiation; (b) gel after exposure to 310 nm radiation.

Knowing that this material exhibits rapid photoisomerization in solution, the same test was performed in the gel state to assess whether this property is preserved, opening the possibility of controlling gelation by using light. When the gel was exposed to 310 nm light, breakdown of the gel was observed due to *E-Z* photoisomerization (Figure 6b). A similar behavior was observed for **3tEST** (Figure S14). When the solution was excited with 365 nm light, no changes were observed. However, when the mixture was heated and then allowed to cool, the gel was formed again.

The formation of molecular gels arises from the initial aggregation of molecules, which subsequently assemble into supramolecular structures capable of intertwining into a three-dimensional network. This network, in turn, immobilizes a large volume of solvent, leading to gel formation.^65,66^ In the case of the molecules studied in this work, this aggregation and supramolecular self-organization process are likely driven by appropriate lateral packing, resulting from van der Waals interactions, π-stacking, and hydrogen bonding. When the sample is irradiated with 310 nm light, the *E-Z* photoisomerization induces a molecular bending that disrupts proper packing and destabilizes the supramolecular structure.^59^ Consequently, this prevents solvent immobilization, ultimately leading to gel breakdown, as observed for these molecules. The reverse isomerization (*Z-E*) using 365 nm light, although effective in dilute solutions, did not restore the gel. This may be due to slow back-isomerization at high concentrations or the material’s difficulty in self-organizing again at room temperature. However, upon heating, thermal relaxation is likely to be favored as well as mobility, facilitating self-organization and explaining the gel restoration.

Thus, although liquid crystalline acylhydrazone gels are known, this work demonstrates that gelation can be reversibly controlled through light exposure, which had not been previously reported for materials with this combination of properties. Consequently, this study introduces new photoresponsive gel-forming materials, contributing to the advancements in the field.

## 3 Conclusions

In summary, we have reported the synthesis and mesomorphic properties of new acylhydrazone-based polycatenars, which differ from each other in the length of the aromatic backbone, number of terminal chains, as well as the orientation and number of the acylhydrazone units. It was demonstrated that each of these features affected the mesomorphic behavior, photoisomerization, and gelation differently. In general, the incorporation of a second acylhydrazone enhanced the materials’ properties compared to similar molecules. The 1,4-bis­(acylhydrazones) derived from the terephthalhydrazine core (**ntHDZ**) displayed greater mesomorphism stability, while the terephthalaldehyde derivatives (**ndHDZ**) displayed a more complex mesomorphism, changing from Col_h_ (**2dHDZ**) to Cub_bi_ with *Ia*3̅*d* symmetry (**3dHDZ**).

The lowest transition temperatures were observed for the smaller molecules, with a higher number of chains, and derived from the terephthalaldehyde core (d-type). This behavior is attributed to weaker intermolecular interactions due to their smaller size, greater packing difficulty caused by steric effects, and potentially less effective hydrogen bonding.

This study further revealed that the sense of the functional group significantly influenced the rate of photoisomerization, with the **ndHDZ**-type favoring this process over the **ntHDZ**-type. Unexpectedly, the **2tHDZ** and **2tEST** molecules did not exhibit photoisomerization in solution, instead displaying slight luminescence. The **2dHDZ** and **3tEST** molecules were able to produce stable gels that could be reversibly undone through photoisomerization. These results highlight the efficiency and versatility of acylhydrazone units in developing LC materials capable of forming different LC phases, offering the potential for a range of applications.

## 4 Experimental Section

### 4.1 Materials and Characterizations

All organic and inorganic reagents and solvents were of the highest purity, purchased from commercial sources (Merck, Sigma-Aldrich, Fluka, Vetec, and Acros Organics), and used as received. The intermediaries 3,4-bis­(dodecyloxy)­benzaldehyde (**3b**),^30^ 3,4,5-tris­(dodecyloxy)­benzaldehyde (**3c**),^67^ 4-formylphenyl 3,4-bis­(dodecyloxy)­benzoate (**4b**),^30^ 4-formylphenyl 3,4,5-tris­(dodecyloxy)­benzoate (**4c**),^30^ 3,4-bis­(dodecyloxy)­benzohydrazide (**5b**)^30^ e 3,4,5-tris­(dodecyloxy)­benzohydrazide (**5c**)^30^ were prepared according to the literature procedures. Anhydrous dichloromethane (CH_2_Cl_2_) was dried using molecular sieves 3 Å for 24 h. Purifications were carried out by recrystallization using commercial grade solvents and by column chromatography on silica-gel 60–200 mesh 60 Å (Merck). Reactions were monitored by thin-layer chromatography (TLC) on aluminum plates coated with a thin layer of silica gel 60 (Merck, Si 60-F254). ^1^H and ^13^C NMR spectra were recorded with a Bruker Avance DRX 400 spectrometer operating at 400 and 100.6 MHz, respectively. Melting points were determined with an Olympus BX53 microscope equipped with a Mettler Toledo FP-82 hot stage.

### 4.2 Thermal Analysis

A polarized optical microscope, an Olympus BX53 coupled to a Mettler Toledo FP-82 Hot Stage, and an Olympus DP73 digital camera were employed to investigate melting point, phase transitions, and mesomorphic textures of all compounds. For the target compounds, thermal transitions and associated enthalpies values were determined by DSC measurements, carried out using a DSC Q2000 calorimeter (TA Instruments) equipped with a RCS90 cooling system, with a heating/cooling rate of 10 °C min^–1^ and a nitrogen flow of 50 mL min^–1^.

### 4.3 X-ray Diffraction

X-ray diffraction (XRD) measurements were performed with an X’Pert PRO (PANalytical) diffractometer using Cu Kα beam (λ = 1.5418 Å), an applied power of 1.2 kVA and using the X’Celerator detector to collect the diffracted radiation. Films were prepared by depositing an amount of powder on a glass plate, where the temperature was controlled with a TCU2000 – Temperature Control Unit (Anton Paar). The scan was carried out in continuous mode from 2 to 30° (2θ angle) with the sample in the mesophase, which was obtained by cooling from the isotropic liquid.

Small-angle X-ray scattering (SAXS) measurements were performed on the SAXS1 beamline at the Brazilian Synchrotron Light Laboratory (LNLS), CNPEM/MCTIC, using a wavelength of 1.544 Å. The samples were placed inside a 1.5 mm quartz capillary (Hampton Research) and inserted into a Linkham Scientific DSC6000 furnace coupled to an LNP95 cooling system. The scattered beam was detected on a Pilatus 300k detector, with a sample-to-detector distance of 823 mm calibrated with silver behenate powder.

With proper indexing from the space group and integrated peak intensities, the electron density (ρ­(*x*, *y*, *z*)) maps were reconstructed (eq 1) via Fourier transform (FT) as

1 $$\rho \left(x,y,z\right)=\sum_{\mathrm{hkl}}\sqrt{I\left(\mathrm{hkl}\right)}\;\exp\left[2\pi i\left(\mathrm{hx}+\mathrm{ky}+\mathrm{lz}\right)+i\phi_{\mathrm{hkl}}\right]$$

For centrosymmetric structures in this paper with electron density ρ­(*x*, *y*, *z*) = ρ­(*x̅*, *y̅*, *z̅*), phase ϕ_*hkl*_ is either 0 or π. This allows an exhaustive approach by comparing all possible phase combinations. The best combination is determined by physical merit of reconstructed electron density (ED) map and other information from the system, like volume ratio of aromatic/aliphatic region and ED distribution histogram.

### 4.4 UV–Vis Spectroscopic Measurements and Photoisomerization

The UV–visible absorption spectra were recorded in a Varian spectrophotometer model, Carry 50Conc, with tungsten and deuterium lamps. The samples were dissolved in spectroscopic chloroform (concentration of 1.0 × 10^–5^ mol L^–1^) and maintained in the dark for 48 h before the spectra were recorded.

The photoisomerization analysis was conducted on the samples described above. Each sample was exposed to 310 nm radiation, with a spectrum recorded every 5 min of exposure until the stationary state (equilibrium between the *E* and *Z* isomers) was reached.

### 4.5 Gelation Properties

In glass test tubes, 4 mg of the material and 1 mL of solvent (toluene, heptane, decane, chloroform, or cyclohexane) were added. Each tube was heated individually until the solution began to boil. The tubes were then allowed to cool while being observed for any signs of precipitation or gelation. See Table S3.

## Supplementary Material



## Notes

Synthetic methodology of intermediates and final products, accompanied by analytical characterization data (melting point, ^1^H and ^13^C NMR, and elemental analysis), TGA and DSC plots, additional POM images, supplementary XRD data, methodology employed for electron density map reconstruction, and additional photophysics, photoisomerization, and gelation data (PDF)
