# Supplementary material for: 1,4-Bis(Acylhydrazone)-Based Polycatenar Liquid Crystals: Self-Assembly, Molecular Switching, and Gelation Properties
Source: ACS Omega. 2025 May 23;10(21):21637–47. doi: 10.1021/acsomega.5c00974 (PMC12138637; doi:10.1021/acsomega.5c00974)
Supplement: Supplementary file 1 [file ao5c00974_si_001.pdf]

Supporting Information

1,4-Bis(Acylhydrazone)-Based Polycatenar Liquid Crystals: Self-Assembly, Molecular Switching and Gelation Properties

Wilson Aparecido de Oliveira[a,b], Mohamed Alaasar[c,d], Yu Cao[e], Eduard Westphal [a,b]*

[a] *Departamento Acadêmico de Química e Biologia, Universidade Tecnológica Federal do Paraná, Curitiba, Brazil.*
[b] *Department of Chemistry, Universidade Federal de Santa Catarina, Florianópolis, Brazil.*
[c] *Institute of Chemistry, Martin Luther University Halle-Wittenberg, 06120 Halle, Germany.*
[d] *Department of Chemistry, Faculty of Science, Cairo University, 12613 Giza, Egypt.*
[e] *Shaanxi International Research Center for Soft Matter, State Key Laboratory for Mechanical Behavior of Materials, Xi'an Jiaotong University, Xi'an 710049, P. R. China*

**corresponding author: eduard.w@ufsc.br;*

Content

**1. Synthesis ……………………………………………………………………………………………………**	**S2**
**2. Termogravimetric analysis curves (TGA) ……………………………………………………**	**S6**
**3. DSC curves ……………………………………………………………………………………………………**	**S7**
**4. Polarized light microscopy micrographs (POM) ……………………………………………**	**S8**
**5. Additional XRD data ………………………………………………………………………………………**	**S8**
**6. Electron density map reconstruction…………………………………………………………**	**S10**
**7. Additional photophysical data ……………………………………………………………………**	**S11**
**8. Gelification ……………………………………………………………………………………………………**	**S13**

1. SYNTHESIS

Terephthalohydrazide (2)

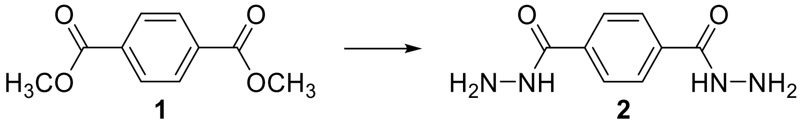

In a 25 mL round-bottomed flask, 2.00 g (10.3 mmol) of dimethyl terephthalate (**1**) and 12.5 mL of toluene were added, and the mixture was heated until it became homogeneous. Then, 5 mL of hydrazine hydrate ($NH_2H_2 \cdot H_2O$, 80%) was added, and the mixture was stirred under reflux for 3 hours. Afterward, the mixture was poured into 100 mL of water, and the resulting suspension was filtered and washed with 50 mL of water and 50 mL of ethanol, yielding 1.58 g (78%) of a white solid. **m.p.** >300 °C. **^{1}H NMR** (400 MHz, DMSO-d$_6$) δ ppm: 7.86 (s, 4H, Ar-H), 9.88 (s, 2H, -CONH-). 13C NMR (101 MHz, DMSO-d$_6$) δ ppm: 127.40, 135.93, 165.59.

N'1,N'4-bis[(E)-3,4-bis(dodecyloxy)benzylidene]terephthalohydrazide (2tHDZ)

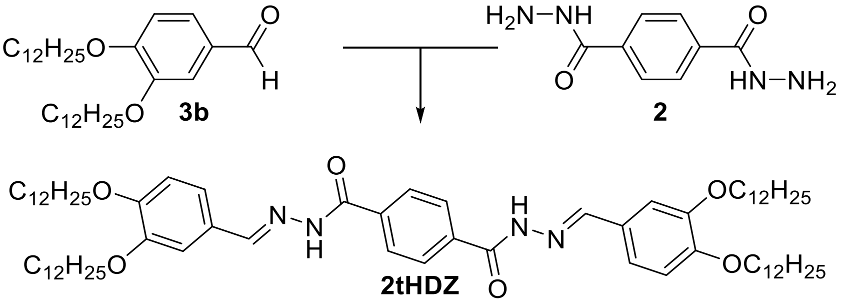

In a 50 mL round bottomed-flask were added 0.10 g (0.51 mmol) of terephthalohydrazide (**2**) and 0.47 g (1.1 mmol) of 3,4-bis(dodecyloxy)benzaldehyde (**3b**). After completely dissolving in 30 mL of hot ethanol, 250 µL of trifluoracetic acid were added to the solution. The reaction was kept under reflux overnight. The reaction was cooled to RT and the precipitate filtered, washed with ethanol then recrystallized multiple times in hot chloroform, affording 0.52 g (94 %) of a white solid. **m.p.** Iso 223 ºC Col$_h$ 195 ºC Cr. **^{1}H NMR** (400 MHz, $C_2D_2Cl_4$) ppm: 0.93 (t, ^3J = 6.7 Hz, 12H, -CH$_3$), 1.27-1.47 (broad signal, 74H, -CH$_2$-), 1.53 (m, 8H, -OCH$_2$CH$_2$CH$_2$-), 1.84 (m, 8H, -OCH$_2$CH$_2$-), 4.07 (t, ^3J = 6.6 Hz, 4H, -OCH$_2$-), 4.08 (t, ^3J = 6.6 Hz, 4H, -OCH$_2$-), 6.93 (dd, ^3J = 8.0 Hz, ^4J = 1.6 Hz, 2H, Ar-H), 7.20 (d, ^3J = 8.0 Hz, 2H, Ar-H), 7.35 (d, ^4J = 1.6 Hz, 2H, Ar-H), 7.99 (s, 4H, Ar-H), 8.23 (s, 2H, -CH=). **^{13}C NMR** (101 MHz, $C_2H_2Cl_4$) δ ppm: 13.88, 22.60, 26.19, 29.29, 29.64, 31.92, 65.47, 70.28, 70.51, 87.51, 99.92, 127.31, 136.73, 150.56, 152.79. **Elemental Analysis** – Calculated for $C_{70}H_{114}N_4O_6$: C, 75.90; H, 10.37; N, 5.06%. Found: C, 76.11; H, 10.45; N, 5.41%.

***N'1,N'4*-bis[(E)-3,4,5-tris(dodecyloxy)benzylidene]terephthalohydrazide (3tHDZ)**

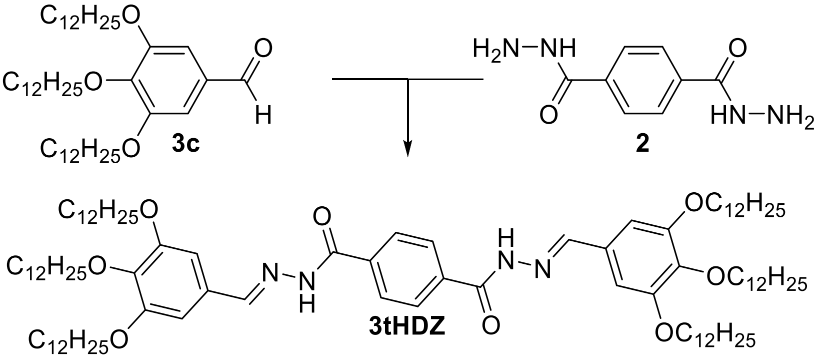

In a 50 mL round bottomed-flask were added 0.10 g (0.51 mmol) of terephthalohydrazide (**2**) and 0.72 g (1.1 mmol) of 3,4,5-tris(dodecyloxy)benzaldehyde (**3c**). After completely dissolving in 30 mL of hot ethanol, 250 µL of acetic acid were added to the solution. The reaction was kept under reflux overnight. The reaction was cooled to RT and the precipitate filtered, washed with water then recrystallized multiple times in hot chloroform, affording 0.41 g (55 %) of a white solid. **m.p.** Iso 183 °C Col_h 146 ºC Cr. **^{1}H NMR** (400 MHz, $CDCl_3$ + drops of DMSO-d_6) ppm: 0.79 (t, ^3J = 6.4 Hz, 18H, $-CH_2C\underline{H}_3$), 1.20-1.33 (broad signal, 96H, $-C\underline{H}_2-$), 1.39 (m, 12H, $-OCH_2CH_2C\underline{H}_2-$), 1.72 (m, 12H, $-OCH_2C\underline{H}_2-$), 3.89 (t, ^3J = 6.6 Hz, 6H, $-OC\underline{H}_2-$), 3.91 (t, ^3J = 6.6 Hz, 6H, $-OC\underline{H}_2-$), 6.90 (s, 4H, Ar-H), 7.97 (s, 4H, Ar-H), 8.27 (s, 2H, -N=CH-), 11.63 (s, 2H, -CONH-). **13C NMR** (101 MHz, $CDCl_3$ + drops of DMSO-d_6) δ ppm: 14.12, 22.59, 26.03, 26.06, 29.26, 29.26, 29.34, 29.50, 29.56, 29.60, 29.65, 30.29, 31.83, 69.09, 73.35, 106.02, 127.97, 129.24, 136.56, 140.10, 149.51, 153.22, 163.57. **Elemental Analysis** – Calculated for $C_{94}H_{162}N_4O_8$: C, 76.47; H, 11.06; N, 3.80%. Found: C, 76.47; H, 11.46; N, 3.80%.

***N',N'''*-[(1E,1'E)-1,4-phenylenebis(methaneylylidene)]bis[3,4-bis(dodecyloxy)benzohydrazide] (2dHDZ)**

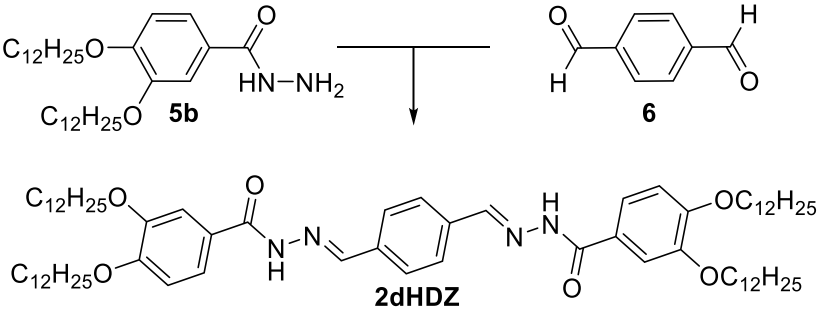

In a 50 mL round bottomed-flask were added 0.067 g (0.50 mmol) of terephthalaldehyde (**6**) and 0.53 g (1.05 mmol) of 3,4-bis(dodecyloxy)benzohydrazide (**5b**). After completely dissolving in 30 mL of hot ethanol, 250 µL of acetic acid were added to the solution. The reaction was kept under reflux overnight. The reaction was cooled to RT and the precipitate filtered, washed with ethanol then recrystallized multiple times in hot ethanol and hot chloroform, affording 0.50 g (90 %) of a white solid. **m.p.** Iso 207 ºC Col_h 192 ºC Cr. **^{1}H NMR** (400 MHz, $C_2D_2Cl_4$) ppm: 0.93 (m, 12H, $-C\underline{H}_3$), 1.27-1.48 (broad signal, 74H, $-C\underline{H}_2-$), 1.53 (m, 8H, $-OCH_2CH_2C\underline{H}_2-$), 1.85 (m, 8H, $-OCH_2C\underline{H}_2-$), 4.10 (t, ^3J = 6.6 Hz, 4H, $-OC\underline{H}_2-$), 4.11 (t, ^3J = 6.6 Hz, 4H, $-OC\underline{H}_2-$), 6.84 (d, ^3J = 8.4 Hz, 2H, Ar-H), 7.48

(dd, ^3J = 8.4 Hz, ^4J = 2.0 Hz, 2H, Ar-H), 7.52 (d, ^4J = 2.0 Hz, 2H, Ar-H), 7.78 (s, 4H, Ar-H), 8.42 (s, 2H, -CH=), 8.84 (broad signal, 2H, -NH-). **Elemental Analysis** – Calculated for $C_{70}H_{114}N_4O_6$: C, 75.90; H, 10.37; N, 5.06%. Found: C, 76.18; H, 10.77; N, 4.95%.

N',N'''-[(1E,1'E)-1,4-phenylenebis(methaneylylidene)]bis[3,4,5-tris(dodecyloxy)benzohydrazide] (3dHDZ)

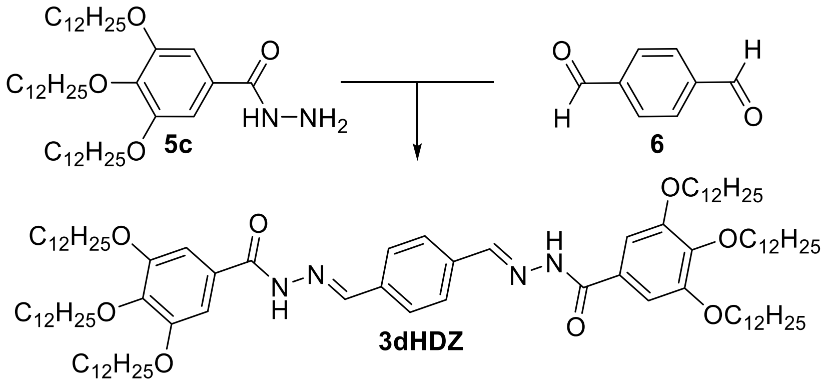

In a 50 mL round bottomed-flask were added 0.067 g (0.50 mmol) of terephthalaldehyde (**6**) and 0.72 g (1.05 mmol) of 3,4,5-tris(dodecyloxy)benzohydrazide (**5c**). After completely dissolving in 30 mL of hot ethanol, 250 µL of acetic acid were added to the solution. The reaction was kept under reflux overnight. The reaction was cooled to RT and the precipitate filtered, washed with ethanol then recrystallized multiple times in hot ethanol, affording 0.52 g (84 %) of a white solid. **m.p.** Iso 165 ºC Cub_{bi} 132 ºC Cr. **^{1}H NMR** (400 MHz, $CDCl_3$ + drops of DMSO-D_6) ppm: 0.88 (t, ^3J = 7.0 Hz, 18H, -$CH_2C\underline{H_3}$), 1.20-1.40 (broad signal, 96H, -$C\underline{H_2}$-), 1.49 (m, 12H, -$OCH_2CH_2C\underline{H_2}$-), 1.82 (m, 12H, -$OCH_2C\underline{H_2}$-), 4.00 (t, ^3J = 6.6 Hz, 6H, -$OC\underline{H_2}$-), 4.04 (t, ^3J = 6.6 Hz, 6H, -$OC\underline{H_2}$-), 7.18 (s, 4H, Ar-H), 7.80 (s.4H, Ar-H), 8.43 (s, 2H, -N=CH-), 11.28 (s, 2H, -CONH-). **^{13}C NMR** (101 MHz, $CDCl_3$) δ ppm: 14.03, 22.56, 26.02, 29.24, 29.30, 29.53, 29.61, 30.24, 31.80, 39.38, 39.59, 39.80, 40.01, 40.22, 40.43, 40.64, 69.20, 73.39, 106.55, 127.66, 135.78, 141.12, 147.51, 152.83, 187.38. **Elemental Analysis** – Calculated for $C_{94}H_{162}N_4O_8$: C, 76.47; H, 11.06; N, 3.80%. Found: C, 76.48; H, 11.41; N, 3.96%.

{(*1E,1'E*)-[terephthaloylbis(hydrazin-2-yl-1-ylidene)]bis(methaneylylidene)}bis(4,1-phenylene) bis[3,4-bis(dodecyloxy)benzoate] (2tEST)

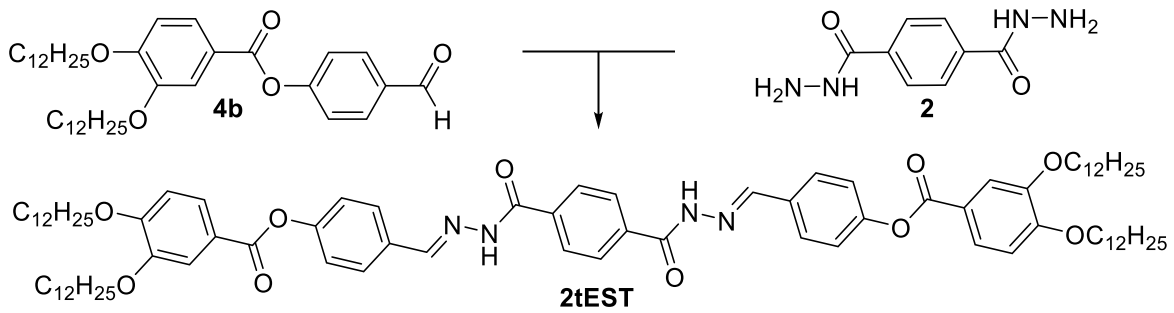

In a 50 mL round bottomed-flask were added 0.05 g (0.25 mmol) of terephthalohydrazide (**2**) and 0.45 g (0.75 mmol) of 4-formylphenyl 3,4-bis(dodecyloxy)benzoate (**4b**). After completely dissolving in 10 mL of hot 1.2-dichloroethane, 0.05 mL of trifluoracetic acid were added to the solution. The reaction was kept under reflux for 1h. The reaction was cooled to RT and the precipitate filtered, washed with ethanol recrystallized multiple times in chloroform, affording 0.18 g (53 %) of a white solid. **m.p.** 267 ºC. **^{1}H NMR** (200 MHz, $CDCl_3$ + drops of TFA-d) ppm: 0.88 (t, ^3J = 6.5 Hz, 12H, $-CH_2C\underline{H_3}$), 1.00-1.20 (broad signal, 64H, $-CH_2-$), 1.45 (m, 8H, $-OCH_2CH_2C\underline{H_2}-$), 1.85 (m, 8H, $-OCH_2C\underline{H_2}-$), 4.16 (t, 8H, $-OC\underline{H_2}-$), 7.06 (d, ^3J = 8.5 Hz, 2H, Ar-H), 7.31 (s, 2H, Ar-H), 7.44 (d, ^3J = 8.4 Hz, 4H, Ar-H), 7.71 (d, ^4J = 2 Hz, 2H, Ar-H), 7.90 (broad signal, 4H, Ar-H), 7.90 (dd, ^3J = 8.4 Hz, ^4J = 2 Hz, 2H, Ar-H), 8.07 (d, ^3J = 8.4 Hz, 4H, Ar-H), 8.87 (s, 2H, -N=CH-), 9.95 (s, 2H, -CONH-). **Elemental Analysis** – Calculated for $C_{84}H_{122}N_4O_{10}$: C, 74.85; H, 9.12; N, 4.16%. Found: C, 74.47; H, 9.18; N, 4.53%.

{(*1E,1'E*)-[terephthaloylbis(hydrazin-2-yl-1-ylidene)]bis(methaneylylidene)}bis(4,1-phenylene)bis[3,4,5-tris(dodecyloxy)benzoate] (3tEST)

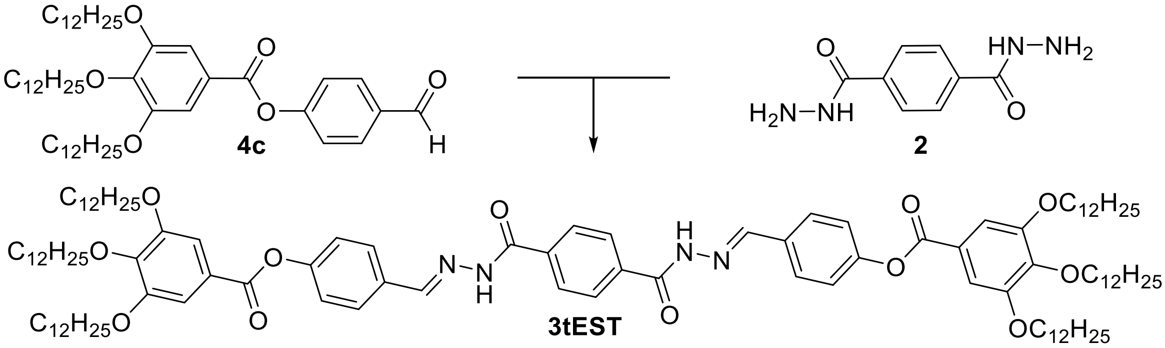

In a 50 mL round bottomed-flask were added 0.10 g (0.5 mmol) of terephthalohydrazide (**2**) and 0.90 g (1.16 mmol) of 4-formylphenyl 3,4,5-tris(dodecyloxy)benzoate (**4c**). After completely dissolving in a hot mixture of 15 mL of t-BuOH and 15 mL of n-BuOH, 0.1 mL of acetic acid were added to the solution. The reaction was kept under reflux overnight. The reaction was cooled to RT and the precipitate filtered, washed with methanol then purified by silica column using dichloromethane: ethyl acetate (95:5) as eluent, and further recovered with hot toluene. Were obtained 0.32 g (37%) of a white solid. **m.p.** Iso 208 ºC Col_h 161 ºC Cr. **^{1}H NMR** (400 MHz, $CDCl_3$) ppm: 0.88 (t, ^3J = 6.4 Hz, 18H, $-CH_2C\underline{H_3}$), 1.00-1.20 (broad signal, 96H, $-CH_2-$), 1.29 (m, 12H, $-OCH_2CH_2C\underline{H_2}-$), 1.59 (m, 12H, $-OCH_2C\underline{H_2}-$), 3.84 (t, ^3J = 6.6 Hz, 6H, $-OC\underline{H_2}-$), 3.85 (t, ^3J = 6.6 Hz, 6H, $-OC\underline{H_2}-$), 7.06 (d, ^3J = 8.5 Hz, 4H, Ar-H), 7.19 (s, 4H, Ar-H), 7.68 (d, ^3J = 8.5 Hz, 4H, Ar-H), 7.88 (s, 4H, Ar-H), 8.30 (s, 2H, -N=CH-), 11.51 (s, 2H, -CONH-). **^{13}C NMR** (101 MHz, CDCl3) δ ppm: 13.96, 22.46, 25.84, 25.88, 29.11, 29.15, 29.40, 29.46, 29.50, 30.13, 31.69, 39.29, 39.50, 39.71, 39.92, 40.13, 40.34, 40.55, 69.03, 73.34, 108.32, 121.98, 123.41, 127.39, 127.62, 127.87, 128.64, 131.80, 142.87, 148.04, 152.35, 152.77, 164.52. **Elemental Analysis** – Calculated for $C_{108}H_{170}N_4O_{12}$: C, 75.57; H, 9.98; N, 3.26%. Found: C, 75.35; H, 9.80; N, 3.38%.

2. TERMOGRAVIMETRIC ANALYSIS CURVES (TGA)

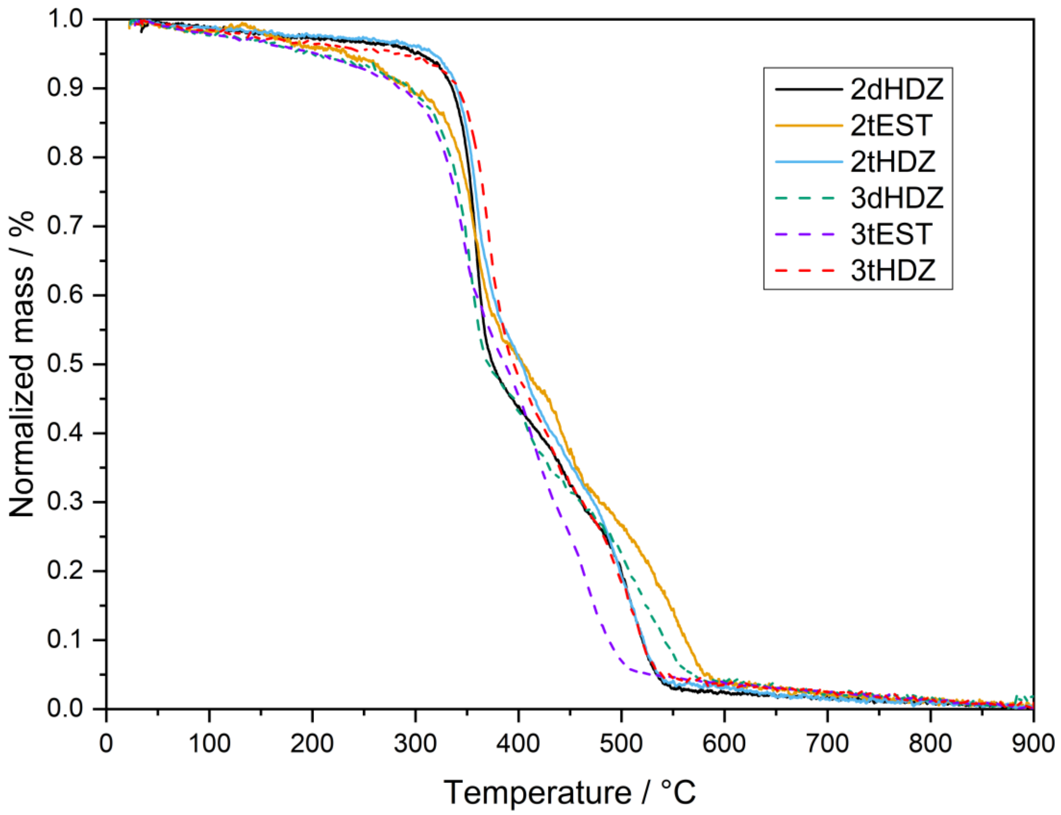

Figure S1. Termogravimetric analysis curves of target compounds (10 °C min^{-1}). **2dHDZ** (black), **2tEST** (orange), **2tHDZ** (cyan), **3dHDZ** (green), **3tEST** (purple), **3tHDZ** (red).

3. DSC CURVES

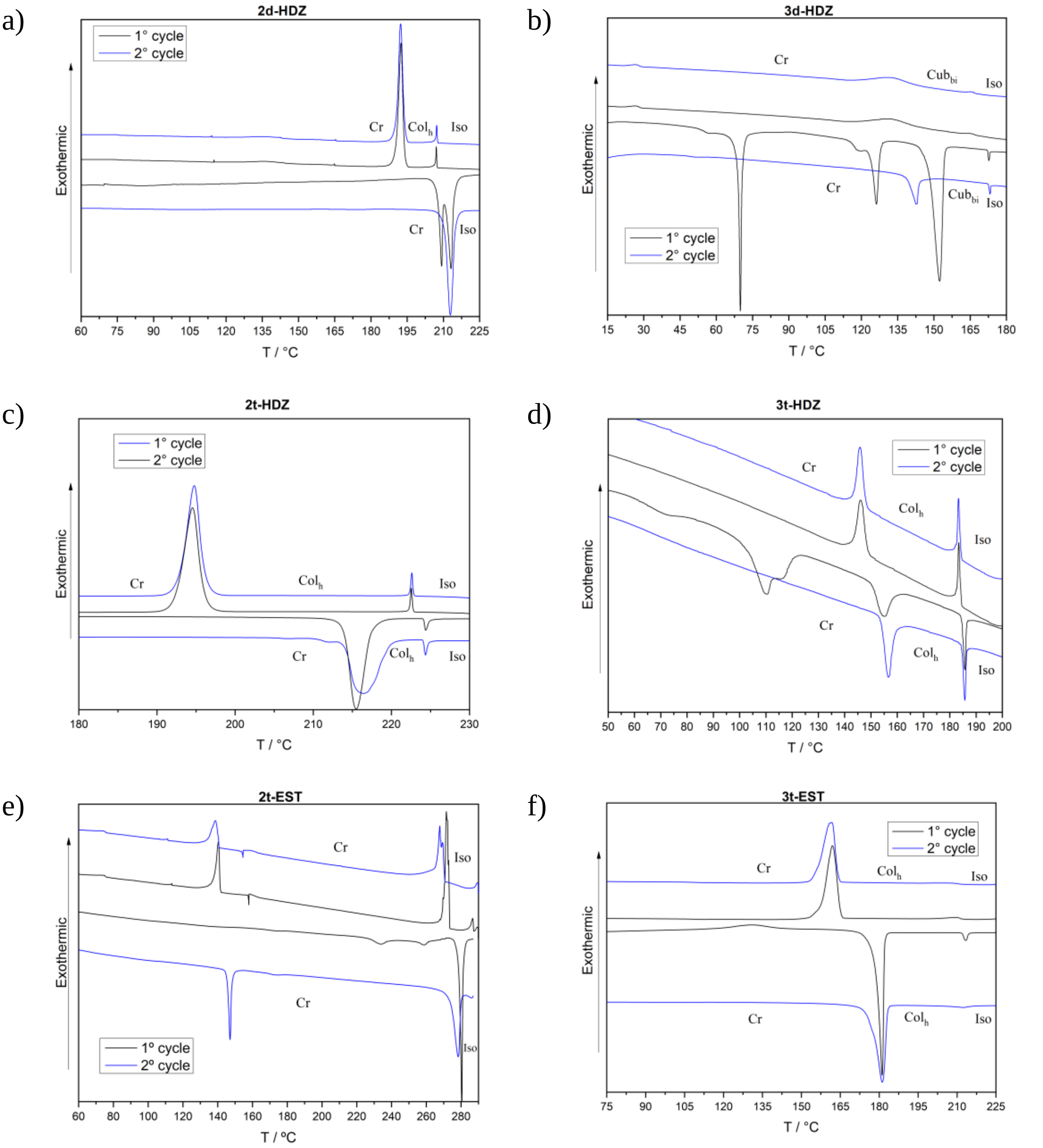

Figure S2. Complete DSC traces for target compounds showing the first (black) and second (blue) heating/cooling cycle (10 °C min^{-1}). a) **2dHDZ** b) **3dHDZ** c) **2tHDZ** d) **3tHDZ** e) **2tEST** f) **3tEST**

Table S1. Transition temperatures, associated enthalpy values and decomposition temperatures for the synthesized compounds

Molecule	T / °C [ΔH / kJ mol^{-1}]^a		T_{dec} / °C ^{b}
	**Heating**	**Cooling**	
**2dHDZ**	Cr **213** [74.2] Iso	Iso **207** [2.5] (Col$_h$) **192** [65.2] Cr	**283**
**3dHDZ**	Cr **143** [5.8] Cub$_{bi}$ **173** [0.4] Iso	Iso **165** [0.4] Cub$_{bi}$ **132** [6.5] Cr	**286**
**2tHDZ**	Cr **216** [99.7] Col$_h$ **224** [2.4] Iso	Iso **223** [2.6] Col$_h$ **195** [108.3] Cr	**306**
**3tHDZ**	Cr **157** [10.6] Col$_h$ **186** [3.2] Iso	Iso **183** [3.5] Col$_h$ **146** [9.1] Cr	**311**
**2tEST**	Cr **147** [21.7] Cr’ **278** [40.7] Iso	Iso **268** [35.0] Cr’ **139** [19.8] Cr	**274**
**3tEST**	Cr **181** [74.5] Col$_h$ **212** [2.8] Iso	Iso **208** [1.2] Col$_h$ **161** [71.6] Cr	**276**

Abbreviations: Cr = crystalline state, Iso = isotropic liquid, Col$_h$ = hexagonal columnar mesophase, Cub$_{bi}$ = Cubic bicontinuous mesophase, () = monotropic phase. ^{a} Determined by DSC (peak temperatures) during the first cooling and second heating cycle using a rate of 10 °C min^{-1}; ^{b} Determined by TGA measurements under nitrogen atmosphere with a heating rate of 10 °C min^{-1}. The values refer to the temperature in which 1% of mass was lost by the material.

4. POLARIZED LIGHT MICROSCOPY MICROGRAPHS (POM)

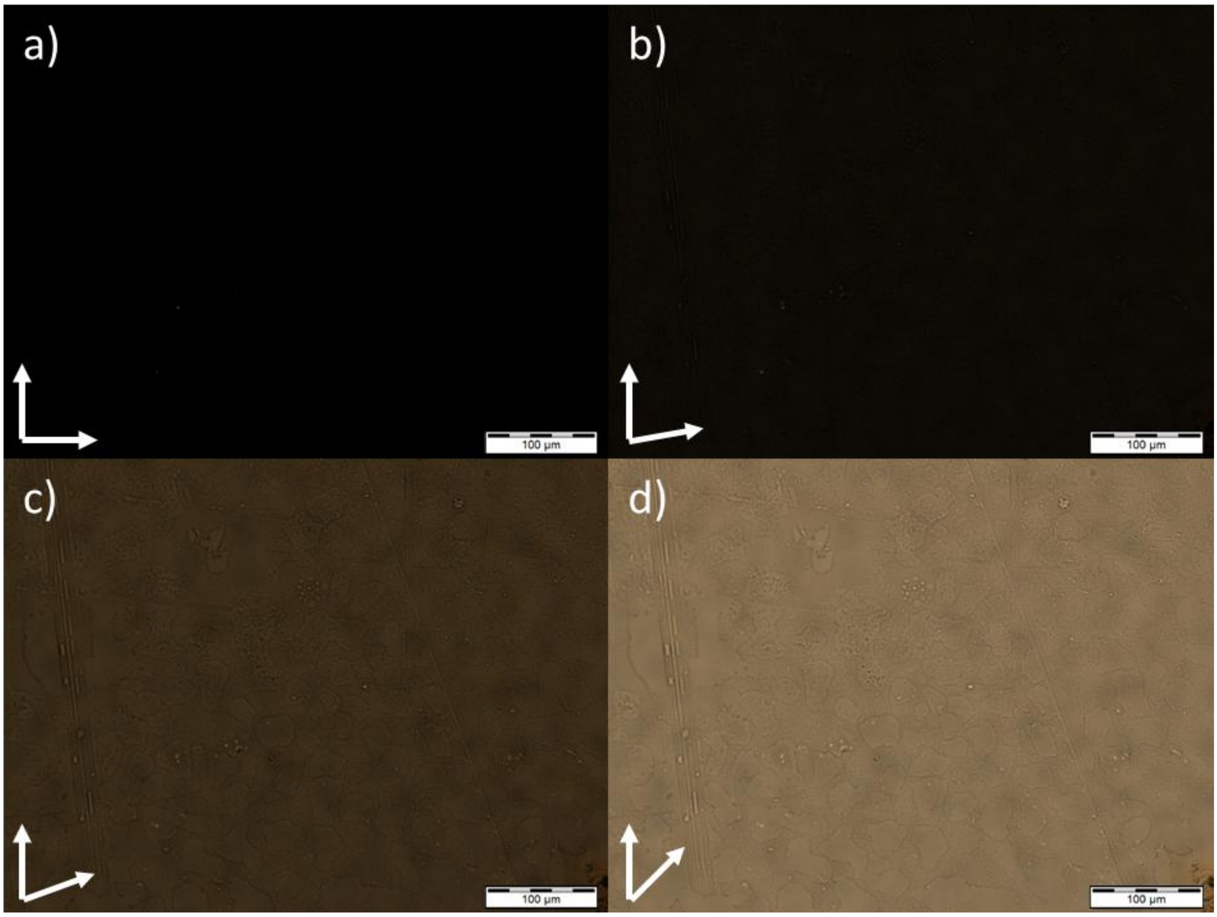

Figure S3. Grooves observed for **3dHDZ** at 150 °C with different polarizers angles. a) 0° ; b) 10° ; c) 20° ; d) 45°.

5. ADDITIONAL XRD DATA

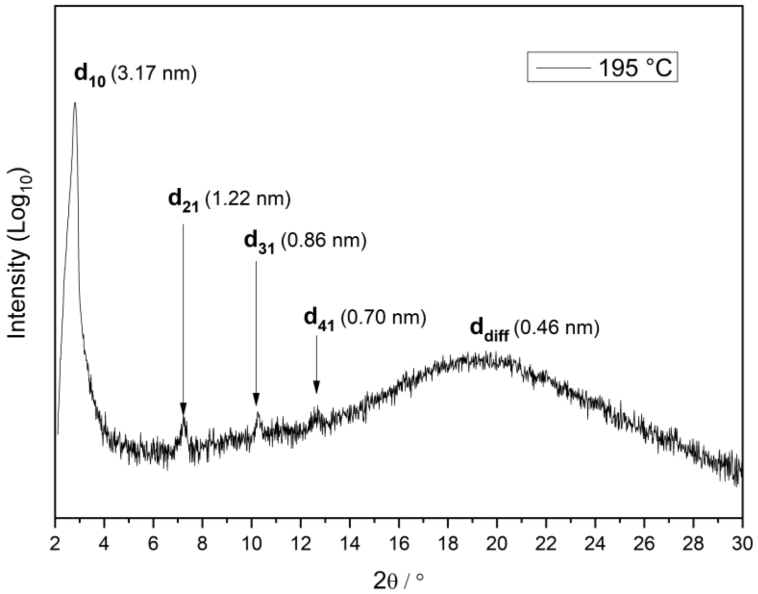

Figure S4. WAXS plot of Col_h of **2tHDZ** at 195 ºC, recorded on cooling from isotropic liquid.

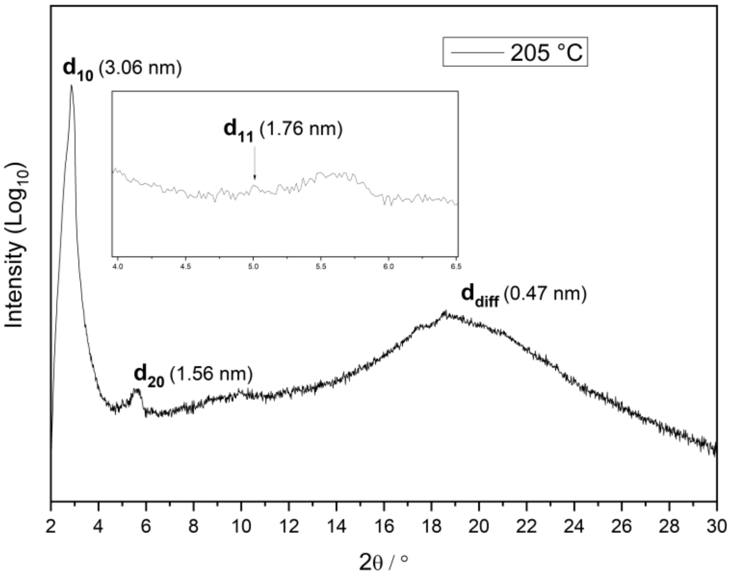

Figure S5. WAXS plot of Col_h of **2dHDZ** at 205 ºC, recorded on cooling from isotropic liquid.

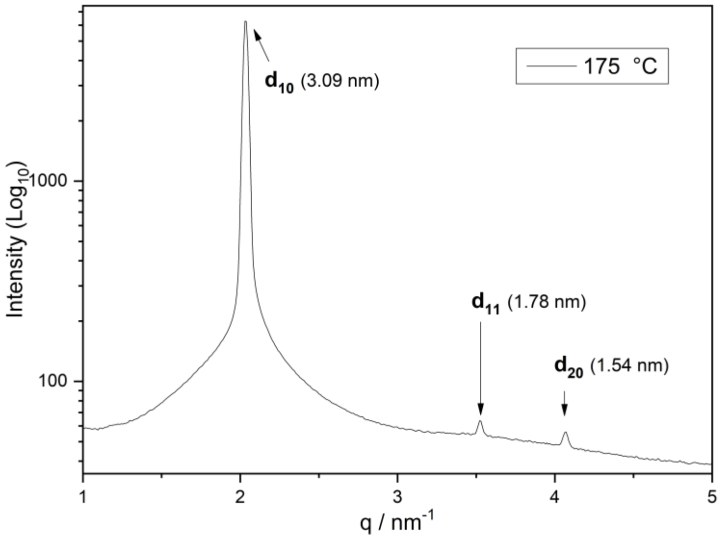

Figure S6. Synchrotron source SAXS plot of the Col_h phase of **3tHDZ** at 175 ºC, recorded on cooling from isotropic liquid.

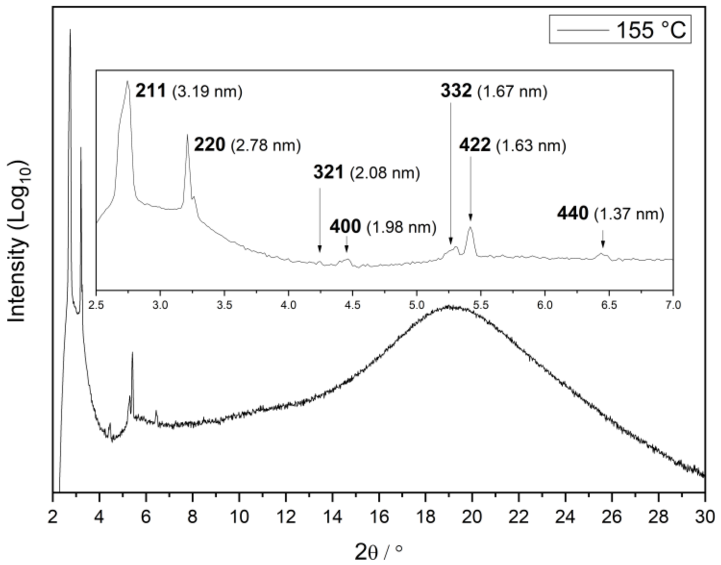

Figure S7. WAXS plot of Cub_{bi} phase of **3dHDZ** at 155 ºC, recorded on cooling from isotropic liquid.

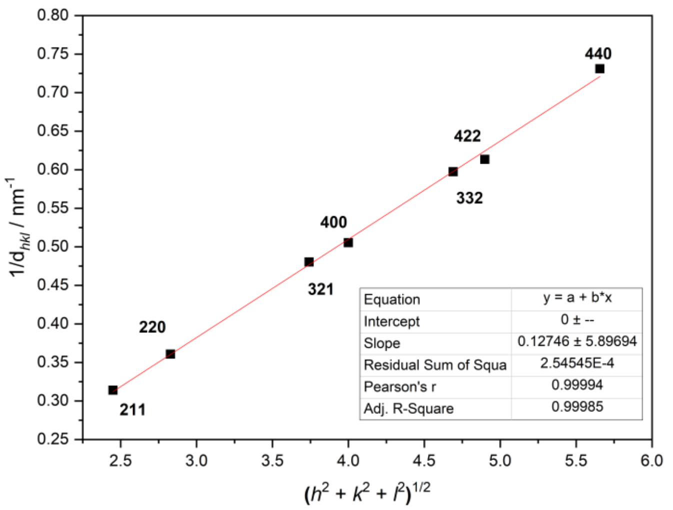

Figure S8. Plot of the inverse of 1/d_{hkl} versus √(h^2+k^2+l^2) for the Cub_{bi} mesophase of the **3dHDZ** product, and linear fit parameters.

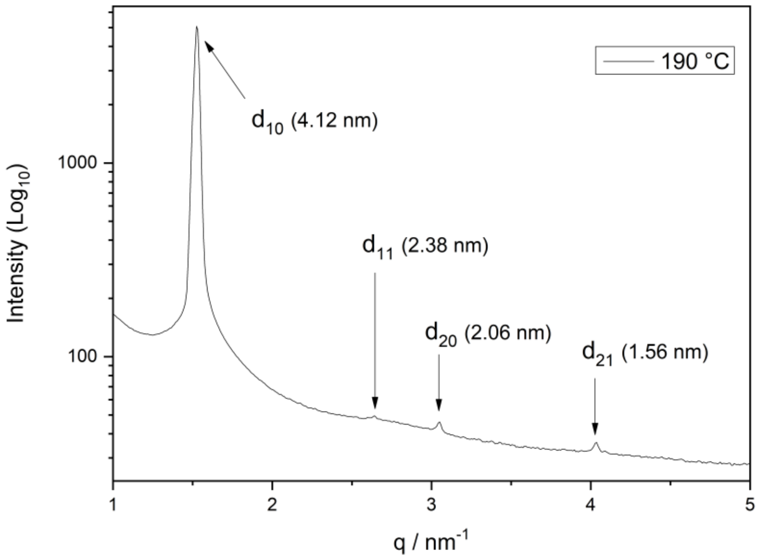

Figure S9. Synchrotron SAXS plot of the Col_h phase of **3tEST** at 190 ºC, recorded on cooling from isotropic liquid.

6. ELECTRON DENSITY MAP RECONSTRUCTION

Once the diffraction intensities are measured, and the corresponding plane group determined by SAXS, 3D electron density maps can be reconstructed based on the general formula.

$$\rho(xyz) = \sum_{hk} F(hkl)\, e^{[i2\pi(hx+ky+lz)]} \qquad (1)$$

Here *F*(*hkl*) is the structure factor of a diffraction peak with index (*hkl*). It is normally a complex number, and the experimentally observed diffraction intensity is

$$I(hkl) = K \cdot F(hkl) = K \cdot |F(hkl)|^2 \qquad (2)$$

Here K is a constant related to the sample volume, incident beam intensity etc. If the constant is equal to 1, then the electron density is

$$\rho(xyz) = \sum_{hkl} \sqrt{I(hkl)}\, e^{[i2\pi(hx+ky+lz)+\varphi_{hkl}]} \qquad (3)$$

As the observed diffraction intensity $I(hkl)$ is only related to the amplitude of the structure factor $|F(hkl)|$, the information about the phase of $F(hkl)$, Φhkl, cannot be determined directly from the experiment. However, the problem is much simplified when the structure of the ordered phase is centrosymmetric; hence, the structure factor $F(hkl)$ is always real, and Φhk is either 0 or π. The case is essentially the same for the 2D centrosymmetric phase.

This makes it possible for a trial-and-error approach, where candidate electron density maps are reconstructed for all possible phase combinations. The “correct” phase combination is then selected on the merit of the maps, helped by prior physical and chemical knowledge of the system. This is especially useful for studying nanostructures, where typically only a limited number of diffraction peaks are observed.

7. ADDITIONAL PHOTOPHYSICAL DATA

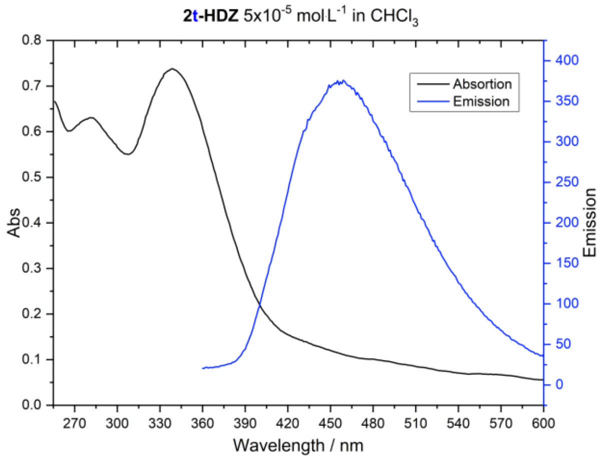

Figure S10. Absorption and emission spectra for compound **2tHDZ** in chloroform solution.

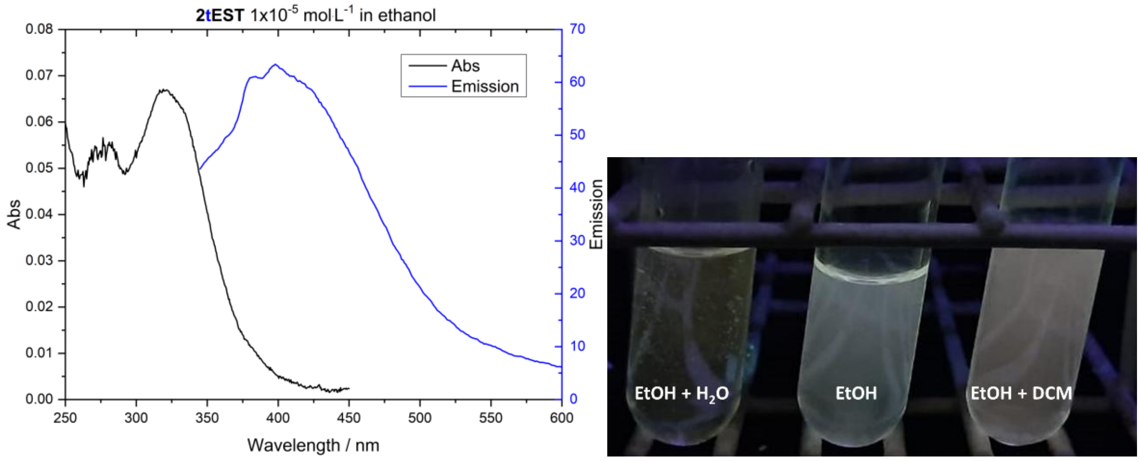

Figure S11. a) Absorption and emission spectra for compound **2tEST** in ethanol solution; b) co-solvent test for **2tEST** emission in ethanol.

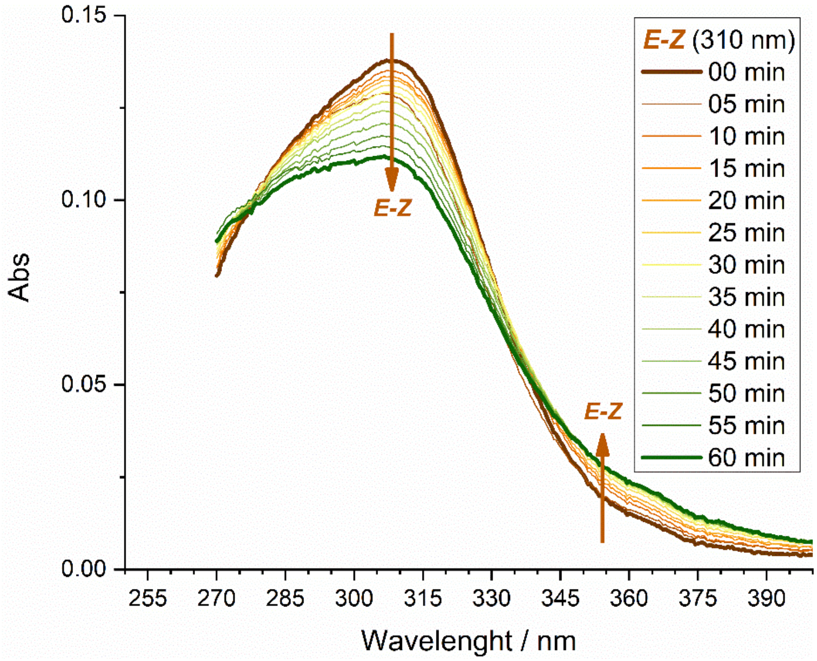

Figure S12. *E-Z* photoisomerization process for the compound **3tEST** using a 310 nm lamp.

Table S2. Photophysical data for target compounds in $CHCl_3$ solution at room temperature (1.0 x 10^{-5} mol L^{-1}).

Compound	λ_{max} / nm	ε / 10^4 L·mol^{-1}·cm^{-1}	λ_{max} Em / nm
**2dHDZ**	346	69	-*
**2tHDZ**	339	74	457
**3dHDZ**	346	53	-*
**3tHDZ**	311	25	-*
**2tEST**	320	6.7	400$^{\#}$
**3tEST**	308	14	-*

λ_{max}: wavelength of maximum absorption *E* isomer; ε: absorption coefficient at λ_{max}; λ_{max} Em: wavelength of maximum emission, with excitation in the region of maximum absorption. * Material did not exhibit luminescence in solution. # measured in ethanol solution.

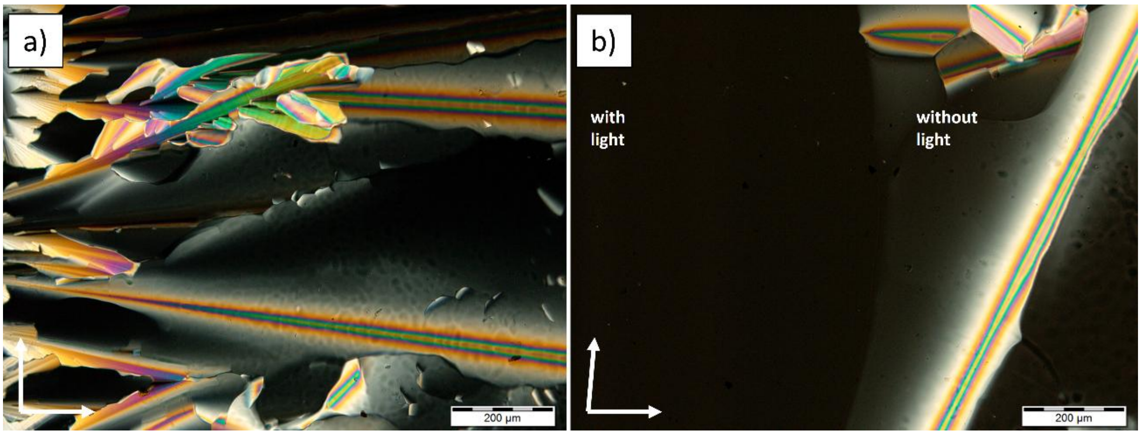

Figure S13. Photoreaction process for the compound **2dHDZ** using a 365 nm lamp (5 W) at 203 °C. (a) Straight linear defects texture before light irradiation, (b) Border between areas exposed or not to UV-light. (direction of polarizers is indicated by white arrows)

8. GELIFICATION

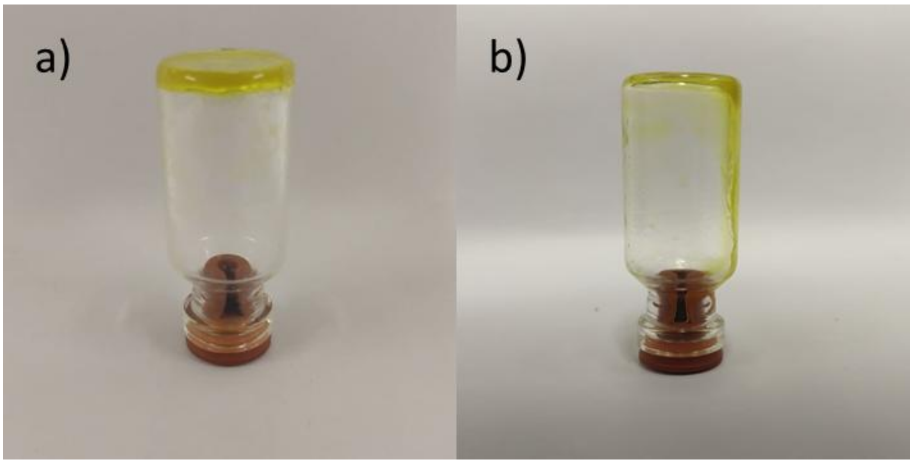

Figure S14. a) gel formed with compound **3tEST** in toluene upon cooling b) gel breaking after long exposure to UV light 310 nm.

Table S3. Gelification tests for target compounds in different solvents at 4 mg·mL^{-1}, determined on cooling from boiling point.

Compound	Solvents				
	**Heptane**	**Toluene**	**Chloroform**	**Cyclohexane**	**Decane**
**2dHDZ**	I	G (white)	I	P	P
**3dHDZ**	S	S	S	G*(white)	S
**2tHDZ**	I	P	I	P	P
**3tHDZ**	S	S	S	S	P
**2tEST**	I	I	I	I	I
**3tEST**	G (white)	G (yellow)	S	I	P

I = insoluble; S = soluble; P = precipitates; G = stable gel (color); G*: unstable gel (color).
